# The ALLPreT panel of legume allergen preparations: characterization of optimized protein extracts for allergen composition, IgE-binding and IgE-crosslinking properties

**DOI:** 10.3389/falgy.2026.1938923

**Published:** 2026-09-28

**Authors:** Ana Laura Olivares-Martínez, Sim Ray Yue, Stefanie Randow, Marie Vestergaard Lukassen, Isabela Assugeni, Maria Oliver, Annette Kuehn, Kitty Verhoeckx, Katrine Lindholm Bøgh, Thomas Holzhauser

**Affiliations:** 1 Division of Allergology, Paul-Ehrlich-Institut, Langen, Germany; 2 National Food Institute, Technical University of Denmark, Kgs. Lyngby, Denmark; 3 Department of Biotechnology and Biomedicine, Technical University of Denmark, Kgs. Lyngby, Denmark; 4 Department of Infection and Immunity, Luxembourg Institute of Health, Esch-sur-Alzette, Luxembourg; 5 Faculty of Science, Technology and Medicine, University of Luxembourg, Esch-sur-Alzette, Luxembourg; 6 InBio, Cardiff, United Kingdom; 7 Department of Dermatology/Allergology, University Medical Center Utrecht, Utrecht University, Utrecht, Netherlands

**Keywords:** allergen extracts, allergenicity assessment, food allergy, IgE, legumes, mass spectrometry, mediator release

## Abstract

**Introduction:**

Allergenicity risk assessment is essential for the authorization of novel foods in accordance with EU Regulation (EU) 2015/2283. Many published studies on the allergenicity assessment of novel foods have been based on study-specific protein preparations with limited comparability across studies. The study aim was to optimize, harmonize and characterize protein extraction from legumes to enable consistent isolation of allergens from relevant protein families in legumes and to be as compatible as possible with *in vitro* cell assays and *in vivo* animal models.

**Methods:**

Various common (peanut, soybean, lupine, pea, lentil, chickpea, white bean, field bean) and small-seeded (fenugreek, alfalfa, red and white clover) legumes were extracted using 4 different buffers at pH ranging from 7.1 to 9.6. The extracts were prepared using suspension, incubation, centrifugation, filtration, and lyophilization processes. Optimized extracts were characterized for their protein profile (SDS-PAGE), protein composition (mass spectrometry), IgG and IgE-binding properties (Western blot), and functional IgE-crosslinking capacity (RBL-2H3 cell mediator release assay, MRA).

**Results:**

Ammonium carbonate buffer (pH 9.0), of which all buffer components fully evaporate during a post-extraction freeze-drying step, resulted in high protein yield with a balanced protein pattern of allergenic proteins within important protein families [11S, 7S, 2S storage proteins; seed albumin, pathogenesis related- 10 (PR-10) protein, profilin, lipid transfer protein, oleosin, defensin, cyclophilin, annexin, seed biotinylated protein, late embryogenesis abundant protein, and others]. All extracts bound IgG from polyclonal peanut or soybean protein antisera, and IgE from legume sensitized subjects. All extracts presented functional IgE-crosslinking properties in legume-specific MRA after passive sensitization with allergen-specific human IgE.

**Discussion:**

The developed procedure harmonizes protein extraction from various legumes. The lyophilized extracts preserve allergenic proteins and, due to lack of buffer components, are compatible for allergenicity testing in both cellular and animal-based models, and diagnostic IgE screening assays. This protocol supports standardization in the allergenicity risk assessment of novel legume foods.

## 1 Introduction

Legumes are considered an important source of protein for human nutrition. They additionally provide dietary fibre, carbohydrates, minerals, vitamins and bioactive compounds (1–3). Besides their nutritional characteristics, legumes have gained interest as an environmentally friendly alternative to animal-food products towards a plant-based diet (2, 4, 5). However, the legume family *Fabaceae* has been associated with food allergic reactions. Peanuts and soybeans are among the major food allergen groups, potentially causing severe adverse reactions. In addition, lupine has been described as cross-reactive to peanut and as a primary sensitizer (6, 7). Despite their general allergenic potential, other legumes like pea, lentil, chickpea, beans and fenugreek appear to present a lower incidence of food allergy (8, 9). Notably, the increasing tendency for plant-based diets to cover the growing demand of dietary products is a driver for the introduction of novel legumes, whose allergenic potential is yet unknown (10–12).

In Europe, the Regulation (EU) 2015/2283 on novel food regulates the authorization of food products that have not been significantly consumed by humans before May 1997. This regulation requires a rigorous assessment to ensure safety, accurate labelling and nutritional adequacy of food products before their introduction into the market (13, 14). Common food allergens are proteins and thus, novel foods containing proteins are considered potentially allergenic (5, 15). Although methods for assessing nutritional, microbiological and toxicological risk have been established, the allergenicity risk assessment of novel foods requires improvement (11, 14, 16, 17).

Current methods for allergenicity assessment of novel foods rely principally on a “weight-of-evidence” approach, following guidelines established by scientific organizations such as the European Food Safety Authority (EFSA), and FAO/WHO including the Codex Alimentarius Commission (14, 16, 18). The existing allergenicity assessment was originally established to evaluate proteins in genetically modified organisms (GMO), focusing on knowledge derived from known food allergens (10, 18). This approach integrates biochemical, bioinformatic and immunological characterization of food proteins. Key elements for the evaluation include sequence homology to known allergens, stability to gastrointestinal digestion and the capacity of the protein to bind Immunoglobulin E (IgE) (11, 14, 15). However, these methods may not be applicable to predict *de novo* sensitization for unknown proteins, therefore, *in vivo* animal testing and *in vitro* cellular models have been proposed to assess potential allergenicity (18, 19). In this context, the EU-funded project ALLPreT aims to develop a robust “Allergenicity Prediction Toolbox for novel foods”, which integrates *in silico*, *in vitro* and *in vivo* models for allergenicity prediction. In experimental allergenicity assessment, suitable protein extracts play a fundamental role. While many published studies on allergenicity assessment of novel foods have relied on study-specific protein preparations, ALLPreT aims to use the same protein preparations from the various study legumes within all *in vitro* and *in vivo* tools to be developed. By integrating the results of the various approaches, this network intends to develop and improve methods to better distinguish between non/weak and strong allergenic proteins. Understanding the differences in legume allergenicity, ALLPreT aims to define a reference set of known allergenic legumes which might be later used as comparators for the assessment of allergenicity of novel legume foods.

Thus, proteins should be extracted efficiently, ideally in a representative and well characterized distribution, and preserving biological activity such as IgE-binding and IgE-crosslinking properties. In previous studies on the optimization of protocols for the extraction of allergenic proteins, the detectability and biological activity, i.e., the quality, of extracted proteins were shown to be influenced by extraction parameters such as buffer composition, pH, ionic strength, and temperature (20, 21).

The objective of this work was to optimize and characterize protein extraction from various dietary legume seeds, for the preparation of legume protein study material that is compatible with *in vitro* immunochemical assays, including diagnostic IgE screening assays, and cellular assays as well as *in vivo* animal tests across all ALLPreT projects. Using in-house available seed materials, four different buffers were evaluated for their protein extraction efficiency, followed by the identification of appropriate extraction conditions to maximize protein recovery while preserving consistent and representative protein profiles in nine different ALLPreT study legumes (peanut, soybean, lupine, pea, lentil, chickpea, white bean, field bean and fenugreek) that were chosen according to their consumption patterns and allergy prevalence in Europe (9, 22). In addition, small-seeded legumes, such as alfalfa and clover (red and white), were included as an example of a potential novel food. Further characterization of the ALLPreT legume protein extracts was done through immunological assays, namely IgE- and IgG-binding and mediator release assays, and relative protein abundance by mass spectrometry.

## 2 Materials and methods

### 2.1 Human plasma samples

Plasma samples were purchased from PlasmaLab International (Washington, USA). These samples were obtained from individuals with suspected or self-reported allergy to peanut, soybean, or other legumes, but without further clinical verification. Upon arrival, the samples were aliquoted and stored at −20 °C to maintain their integrity and to prevent multiple freeze-thaw cycles. For the measurement of sIgE against legumes, samples were analysed using the ALEX^2^ Allergy Xplorer multiplex method (MacroArray Diagnostic GmbH, Vienna, Austria), an immunoassay test based on Enzyme-Linked Immunosorbent Assay (ELISA), following the manufacturer's instructions. Analysis of the developed arrays was done with the RAPTOR SERVER Analysis software 1.22.3. The sIgE levels of serum samples against legumes are summarized in Supplementary Table S1.

### 2.2 Seed material

#### 2.2.1 Seed material used for optimization of protein extraction protocol

In-house available dry legume seeds from different local distributors were used to compare protein profiles obtained from different extraction buffers. This material (Table 1) was not included in further analysis within the ALLPreT projects.

**Table 1:** In-house legume seed material specifically used for the selection of extraction buffer.

Seed name	Cultivar	Company
Roasted peanut (*Arachis hypogaea*)	Jumbo giant	A
White lupine (*Lupinus albus*)	Fedora	B
Yellow lupine (*Lupinus luteus)*	Mister ZS	C
Blue lupine (*Lupinus angustifolius)*	Bora	D
Yellow soybean (*Glycine max*)	—	E
Pea *(Pisum sativum)*	Regina	F
Green pea (*Pisum sativum*)	—	G
Brown lentil (*Lens culinaris*)	Plate lentils	G
Red lentil (*Lens culinaris*)	—	H
Chickpea (*Cicer arietinum*)	—	H
White bean (*Phaselus vulgaris*)	—	I
Kidney bean (*Phaselus vulgaris*)	—	H
Pinto bean (*Phaselus vulgaris*)	—	H

The seeds listed were not included in any subsequent optimization steps. Seeds were purchased from or provided by: A: Seeberger; Germany; B: Südwestdeutsche Saatzucht; Germany; C: Feldsaaten Freudenberger; Germany; D: Saatzucht Steinach; Germany; E: Hensel, W. Schoenenberger; Germany; F: Saatzucht Aschersleben; Germany; G: Müller's Mühle; Germany; H: Rapunzel Naturkost; Germany; I: Delikatesse; Germany. (— unknown cultivar).

#### 2.2.2 Seed material used within ALLPreT project

After consensus within the ALLPreT consortium, legume seeds were selected based on their use and consumption as protein sources, ensuring inclusion of some legumes with high (e.g., *Arachis hypogaea*) to low (e.g., *Phaseolus vulgaris*) reported allergy prevalence. Legumes were classified as common or small-seeded legumes according to seed characteristics. Dry seeds were purchased from different local distributors, as summarized in Table 2, and stored at room temperature in a dark and dry place.

**Table 2:** ALLPreT legume seed material.

Seed name	Cultivar	Company
Common legumes		
Roasted peanut (*Arachis hypogaea*)	Jumbo	A
Unroasted peanut (*Arachis hypogaea*)	High Premium	A
Sweet blue lupine (*Lupinus angustifolius*)	Rumba	B
Soybean (*Glycine max*)	ES Comandor	B
Winter pea (*Pisum sativum*)	E.F.B. 33	B
Beluga lentils (*Lens culinaris*)	—	B
Chickpea (*Cicer arietinum*)	KIRA	C
Field bean (*Vicia faba*)	Bobas	B
White kidney bean (*Phaseolus vulgaris*)	—	D
Small-seeded legumes		
Fenugreek (*Trigonella foenum-graecum*)	—	B
Alfalfa (*Medicago sativa*)	Giulia	B
Red clover (*Trifolium pratense*)	Vytis	B
White clover (*Trifolium repens*)	Rivendel	B

Seeds were purchased from: A: Paul's Mühle Fachmarkt & Onlineshop für Tier, Garten und Naturkost, Germany; B: Camena Samen Inhaber Marc-Philip Steg e.K., Germany; C: P.H. Petersen Saatzucht Lundsgaard, Germany; D: KoRo Handels GmbH, Germany. (— unknown cultivar).

### 2.3 Legume seed total protein extraction

#### 2.3.1 Extraction buffers

Four different buffers were selected to compare the extraction performance regarding quality (protein composition) and quantity (protein yield) (Table 3). Tris-buffered saline (TBS) and Phosphate-buffered saline (PBS) were included as commonly used buffers for protein extraction and testing (23, 24). Ammonium carbonate (AC) and sodium carbonate (SC) buffers were included to determine the effects of high pH on protein extraction. The most optimal buffer was later selected based on protein profile and yield, and potential compatibility with cellular assays and animal experiments.

**Table 3:** Buffers used for legume seed protein extraction.

Buffer	pH	Composition
Tris-buffered saline (TBS)	7.4	50 mM Tris-HCl; 150 mM sodium chloride
Phosphate-buffered saline (PBS)	7.1	137 mM sodium chloride; 2.7 mM potassium chloride; 8 mM sodium phosphate dibasic; 1.5 mM potassium phosphate monobasic
Ammonium carbonate (AC)	9.0	100 mM ammonium carbonate (NH_4_)_2_CO_3_
Sodium carbonate (SC)	9.6	50 mM sodium carbonate Na_2_CO_3_

#### 2.3.2 General procedures for extract preparation

Roasted and unroasted peanuts were deshelled and dehulled; the other legume seeds were applied without pretreatment. All dry legume seeds were frozen using liquid nitrogen, and grinded to a fine powder using a cryogenic mill (CryoMill, Retsch, Germany) applying three grinding cycles at a frequency of 30 Hz for 1 min each, followed by an intermediate cooling step at 5 Hz for 30 s. The resulting powder was stored at −80 °C until use.

Protein extracts were prepared by suspending frozen seed powder in extraction buffer, followed by incubation, centrifugation, filtration and finally lyophilization. Specific extraction parameters were applied during optimization and are detailed in the section “Optimization of legume protein extraction”.

Additionally, extracts were centrifuged at 16,000–20,000 *g* for 30–40 min at 4–10 °C. The resulting supernatants were filtered through 5 µm, 1.2 µm and 0.2 µm Minisart® NML Cellulose Acetate (hydrophilic) (Sartorius AG, Germany) or Polyethersulfone (PES) (Carl Roth, Germany) filters. Aliquoted (1 mL or 5 mL) extracts were stored at −80 °C. The extract aliquots were freeze-dried overnight in a lyophilizer (Alpha 2–4 LSCplus, Martin Christ Gefriertrocknungsanlagen, Germany) and stored at −20 °C until use.

#### 2.3.3 Optimization of legume protein extraction

The overall process for optimization of protein extraction is schematically represented in Figure 1.

**Figure 1:**
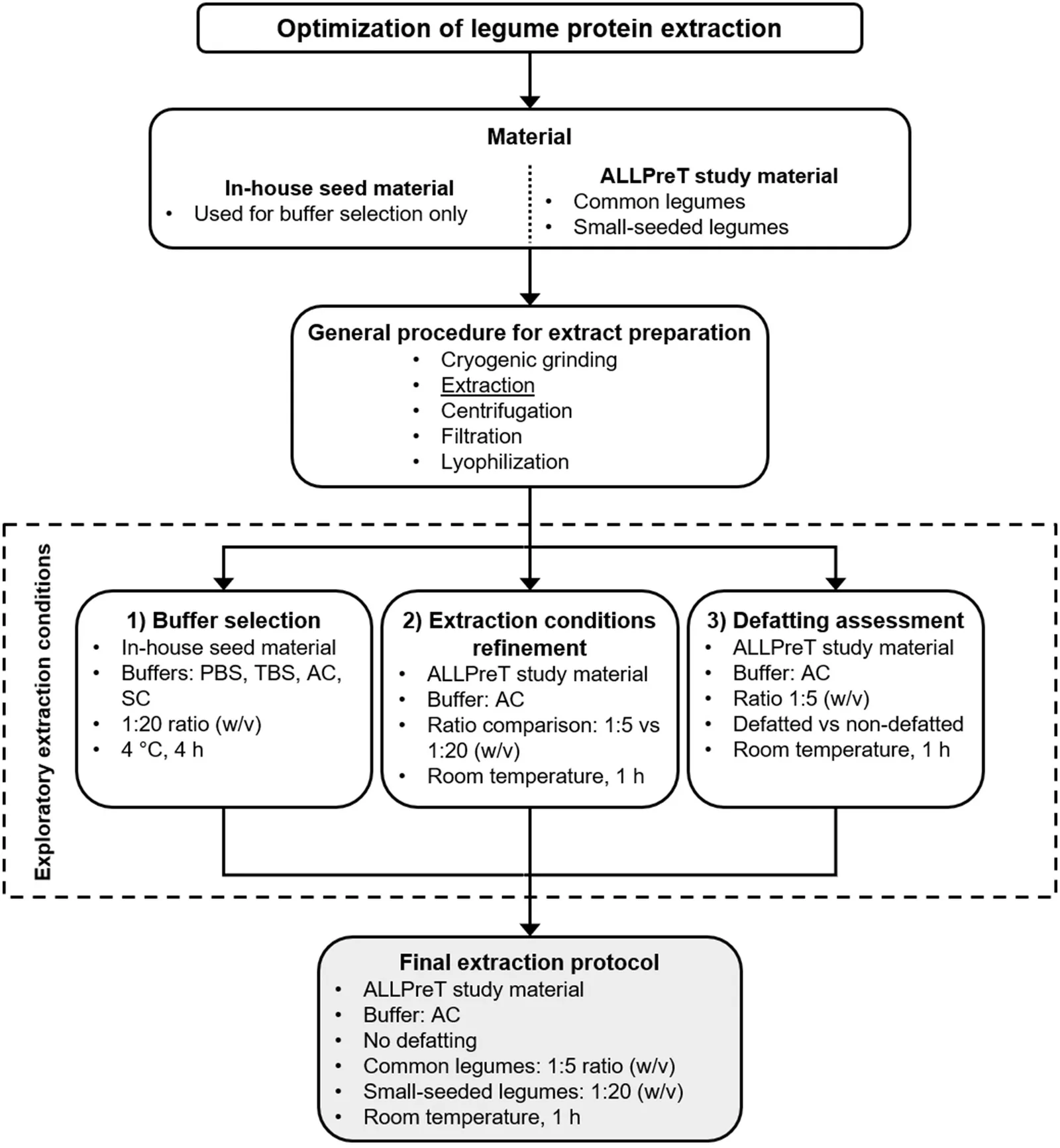
Workflow for the optimization of extraction conditions for legume total protein extracts. The buffer selection (Table 3) was performed with the in-house available legume seed material (Table 1) under identical extraction conditions (extraction ratio, temperature, time). Extraction conditions, including evaluation of defatting, on extraction performance were refined using ALLPreT study material. The final protocol was applied using AC buffer without defatting, with extraction ratios according to legume classification. (w/v, weight per volume).

Starting with the in-house legume seed material (Table 1), legume proteins were extracted at a 1:20 (w/v) ratio vs. respective extraction buffer. Samples were vortexed continuously (VWR 444-1372, VWR International, Germany) until a complete homogenous suspension was obtained and subsequently incubated on constant agitation in a rotator (Stuart SB3—Rotator, Bibby Scientific, UK) at 15 rpm for 4 h at 4 °C. The extraction conditions were refined on the subsequent use of the ALLPreT study material (Table 2, Figure 1).

The effect of defatting on protein extraction was investigated in a selection of ALLPreT legume seeds (roasted peanut, unroasted peanut, sweet blue lupine, soybean, winter pea, beluga lentil chickpea, field bean). Seed powder was defatted using n-hexane (Carl Roth, Germany) at a ratio of 1:5 (w/v) with continuous agitation for 1 h at room temperature. The solvent and solid phases were separated by gravity filtration through pleated filter paper (Whatman, Grade 595 via Merck, Germany). The defatting step was repeated twice and at the last step, the residue was air-dried overnight in a fume hood to remove residual n-hexane, and the defatted powder was stored at −80 °C until use. Defatted seed material was extracted as described above (Figure 1).

#### 2.3.4 Optimized extraction protocol using ammonium carbonate buffer

ALLPreT legume seeds were ground in liquid nitrogen as described above. Total protein extracts were obtained by resuspending either 0.25 or 1 g of non-defatted frozen seed powder into 100 mM AC buffer (pH 9.0). The buffer was added in a ratio of 1:5 (w/v) for the common legumes and 1:20 (w/v) for the small-seeded legumes, and samples were vortexed to obtain a homogeneous suspension. The extraction was carried out at room temperature on constant agitation in a rotator at 15 rpm for 1 h. Extracts were centrifuged, filtrated, frozen, lyophilized, and stored as described above under section “General procedures for extract preparation”. Prior to further analysis, lyophilized total protein was restored in water, or PBS for MS analysis.

### 2.4 Protein quantification and estimation of extraction efficiency

Per 1 mL of lyophilized extract, aliquots were restored in 1 mL deionized water. Samples were carefully vortexed until all protein appeared to be solved. Protein concentration was determined in duplicates against Bovine Serum Albumin (BSA, Sigma-Aldrich, Germany) as a standard, using a commercial Bradford method according to the manufacturer's instructions (Roti®-Nanoquant; Carl Roth, Germany).

Reference values of total protein of legume seeds were taken from publicly available compositional databases and literature. Where possible, crude protein values based on nitrogen determination (*N* × 6.25) were used; for species lacking such data, protein values derived from amino acid analysis reported in the literature were used as reference estimates.

Estimates of extraction efficiency were calculated as the ratio between the total extracted protein (mg protein per g of starting material) and the reference protein content, expressed as percentages:

$$\mathrm{Extraction}\;\mathrm{efficiency}\;\;(\text{\%})=\;\frac{\mathrm{Total}\;\mathrm{extracted}\;\mathrm{protein}\;\;(\text{\%})}{\mathrm{Reference}\;\mathrm{protein}\;\mathrm{content}\;(\text{\%})}\times 100$$

### 2.5 SDS−PAGE

Total protein extract was separated using SDS-PAGE according to the method of Laemmli (25) with 13% resolving and 5% stacking gels. Under reducing conditions, samples were diluted in sample buffer [0.2% SDS, 10% glycerine, 62.6 mM Tris HCl pH 6.8, 100 mM Dithiothreitol (DTT), 0.01% Bromophenol blue (w/v)] and heated at 95 °C for 10 min and loaded to 1.0 mm thick gels (BioRad MiniProtean 3 cell, Germany). For samples under non-reducing conditions, the sample buffer did not contain DTT. For subsequent Coomassie-staining, 10 µg of total protein were loaded per cm length of gel pocket. For subsequent immunoblot analysis, 20 µg/cm were loaded.

For comparison of molecular weight, 3 µL of low molecular weight marker were added (LWM marker, Cytiva, Germany), and gels were run at 150–180 V until bromophenol blue reached the bottom of the gel. Gels were either Coomassie-stained for total protein visualization or blotted onto membrane for immunoblot analysis.

### 2.6 Immunoblot analysis with animal IgG and human IgE

Proteins separated in SDS-PAGE were semi-dry blotted to nitrocellulose membranes (pore size 0.2 µm, GE Healthcare, Germany) at 0.8 mA/cm^2^ for 1 h. The transfer buffer used was 25 mM Tris, 20% Methanol, pH 10.4. Transferred membranes were stained with 0.1% Ponceau S in 5% acetic acid solution for 10 min. Membranes were cut into strips of 0.3 cm width, destained with TBS and blocked twice for each 30 min with 0.3% Tween 20 in TBS, followed by two washes of 5 min each with 0.05% Tween 20 in TBS (TBST). After each incubation with primary or secondary antibody, the membrane strips were washed five times for 5 min with TBST. Membrane strip development was performed using nitro blue tetrazolium (NBT, Sigma-Aldrich, Germany) and 5-bromo-4-chloro-3-indolyl phosphate (BCIP, Sigma-Aldrich, Germany) prepared in TBS buffer. Reactions were stopped after 2–20 min, depending on band intensity.

IgG-immunostaining of total protein extracts from defatted and non-defatted peanuts was done using rabbit anti-peanut polyclonal antibody (pAb) (26) and monoclonal antibodies (mAb) targeting Ara h 1 (Pn-t) (26), Ara h 3 (PEI-7B2), Ara h 2 and Ara h 6 (1C4 and 3B8 respectively (InBio, Charlottesville, USA). The pAb and mAb were diluted in incubation buffer (0.1% BSA in TBST) as follows: anti-peanut pAb at two dilutions 1:60,000 and 1:10,000, anti-Ara h 1 and Ara h 2 at 1:10,000, anti-Ara h 3 at 1:1,000 and anti-Ara h 6 at 1:2,000. Strips were incubated overnight at 4 °C. Detection was made with alkaline phosphatase (AP)-labelled mouse anti-rabbit IgG (Sigma-Aldrich, Germany) diluted 1:5,000, and goat anti-mouse (Sigma-Aldrich, Germany) diluted 1:10,000 in incubation buffer, and incubated for 90 min.

IgG cross-binding among legume species was assessed with polyclonal anti-soybean (Riedel, R-Biopharm, R45254, Darmstadt, Germany) and anti-peanut (26) rabbit antibody. The anti-soybean antibody was applied at dilution of 1:100,000 (common legumes) and 1:25,000 (small-seeded legumes), while the anti-peanut antibody was used at a dilution of 1:10,000 for all legumes. Membrane strips were incubated overnight at 4 °C. Bound IgG was detected with AP-labelled mouse anti-rabbit IgG (Sigma-Aldrich, Germany) diluted 1:10,000 in incubation buffer, and incubated for 90 min.

Human sera as source of allergen-specific IgE were diluted at 1:20 in incubation buffer, and membrane strips were incubated overnight at 4 °C. Bound IgE was detected with AP-labelled mouse anti-human IgE (Southern Biotech via Biozol, Germany) diluted 1:10,000 in incubation buffer, and incubated for 90 min.

### 2.7 Mediator release assay (MRA)

Mediator release followed a protocol previously described (27) with some modifications. Rat basophilic leukemia (RBL)-2H3 cells transfected with the Fc*ε*RI *α*-chain of human IgE receptor were incubated overnight with human sera diluted 1:20 (Minimal Essential Medium (MEM, Gibco via Thermo Fisher, Scotland), 5% [v/v] fetal calf serum (FCS, Sigma Aldrich, Germany), 1% [v/v] L-glutamine) for sensitization. Sensitized cells were then stimulated with increasing concentrations (100 pg/well–30 µg/well) of each total legume seed extract at a total volume of 100 µL/well. To determine total release, passively sensitized cells were lysed with 1% Triton X-100 (Sigma-Aldrich, Germany). *β*-hexosaminidase activity in the supernatant was measured spectrophotometrically (SpectraMax ABS, Molecular Devices GmbH, Germany) at a wavelength of 405–620 nm. Results were analysed with the SoftMax Pro 7.1 software (Molecular Devices) and expressed as a percentage of the total release after correction for buffer baseline release. For each serum, a serum release control in the absence of legume extract was run on each plate, and subtracted from each allergen-specific release. In addition, for the three highest protein concentrations, triplicate antigen release controls in the absence of serum were run for each legume extract, and subtracted from each allergen-specific release. As all release data were corrected for serum and antigen controls, the processed data reflect legume protein-specific release only.

Based on basophil responses, the tested serum (*n* = 12) was divided into two groups: reactive and non-reactive. Threshold cut-off for positive release was calculated from the reactive group (*n* = 8) as the mean percentage of spontaneous release of passively sensitized but non-stimulated cells (serum control without antigen) plus two standard deviations. Calculated threshold was confirmed, following a similar approach as reported by Stoffersen, et al. (28), by calculating the means from the stimulated non-reactive group (*n* = 4) of all dilution points plus two standard deviations. Responses above the cut-off (10% of total release) were considered positive.

### 2.8 Determination of relative protein abundance by LC-MS/MS

Lyophilized extracts were resuspended with PBS and enzymatically digested. The resulting peptides were subjected to liquid chromatography-tandem mass spectrometry (LC-MS/MS) using the Orbitrap Exploris™ 480 mass spectrometer (Thermo Scientific™) coupled to the EASY-nLC 1200 LC system (Thermo Scientific™). Acquired spectra were analysed using Spectronaut® 20 (Biognosys) and searched against the corresponding taxonomy in the UniProt database. The relative protein abundance was estimated using intensity-based absolute quantification (iBAQ)-derived values, where the iBAQ value of a given protein group was expressed as a percentage of the summed iBAQ values of all quantified protein groups. The resulting abundances were used for identification and quantification of both proteins belonging to allergen-related protein families and proteins not belonging to allergen-related protein families. Protein identifications were filtered at a false discovery rate of 1% and protein quantitation was performed at the MS1 level. Detailed experimental conditions, database search parameters, and protein identification criteria are provided in the Supplementary material (Supplementary methods).

### 2.9 Endotoxin measurement

Endotoxin was quantified using the LAL kinetic turbidimetric assay according to Remillard et al. (29). Appropriate extract dilution was verified by spike recovery between 50% and 200%. Thus, extracts were finally measured at dilutions of 1:100, results corrected for the dilution factor and expressed as EU endotoxin per mg extracted protein. Control analysis using Glucashield (Associates of Cape Cod Incorporated, Falmouth, MA), that blocks interference by (1 → 3)-ß-glucans from cellulosic materials or other glucan contamination from fungal or bacterial origin, indicated no potentially false positive results due to (1 → 3)-ß-glucans.

### 2.10 Statistical analysis

Statistical analysis was done using GraphPad Prism Software (version 9.5.0 for Windows, San Diego, California, USA). Differences in the amount of extracted protein using different buffers were examined by analysis of variance (ANOVA) followed by the Tukey-test for the comparison of the means. Differences between defatted and non-defatted samples were analysed using a paired Student's *t*-test. The differences were considered significant at *p* < 0.05.

## 3 Results

### 3.1 Ammonium carbonate buffer allows good yield and balanced composition of proteins extracted from legume seeds

Four different buffers (Table 3) were assessed for their performance in protein extraction from in-house legume seed material (Table 1) under the same conditions. The buffers were selected based on their potential compatibility with both *in vitro* antibody assays and biological cellular assays, as well as with *in vivo* models. Resulting protein concentration and electrophoretic profiles were compared between the extracts after lyophilization and reconstitution with pure water. Protein concentration (Figure 2A, Supplementary Table S2) varied among legumes extracted with the different buffers, although the obtained values were consistent within seeds from the same species. Both carbonate buffers with high pH were able to extract a significantly higher amount of total protein in comparison to buffers with lower pH (Figure 2B), for example, for roasted peanut and yellow lupine, where the extracts prepared with AC and SC were able to extract more than double the amount than those prepared with TBS and PBS (Figure 2A, Supplementary Table S2). Protein concentrations obtained with AC were significantly higher than with TBS or PBS (*p* < 0.001). Similarly, SC buffer also extracted significantly higher protein amounts than TBS (*p* < 0.05) or PBS (*p* < 0.01). On average, AC buffer showed the highest extraction yield. However, the statistical analysis showed no significant difference between AC and SC buffers (mean 5.8 and 5.2 mg/mL) or between TBS and PBS (mean 3.4 and 2.8 mg/mL).

**Figure 2:**
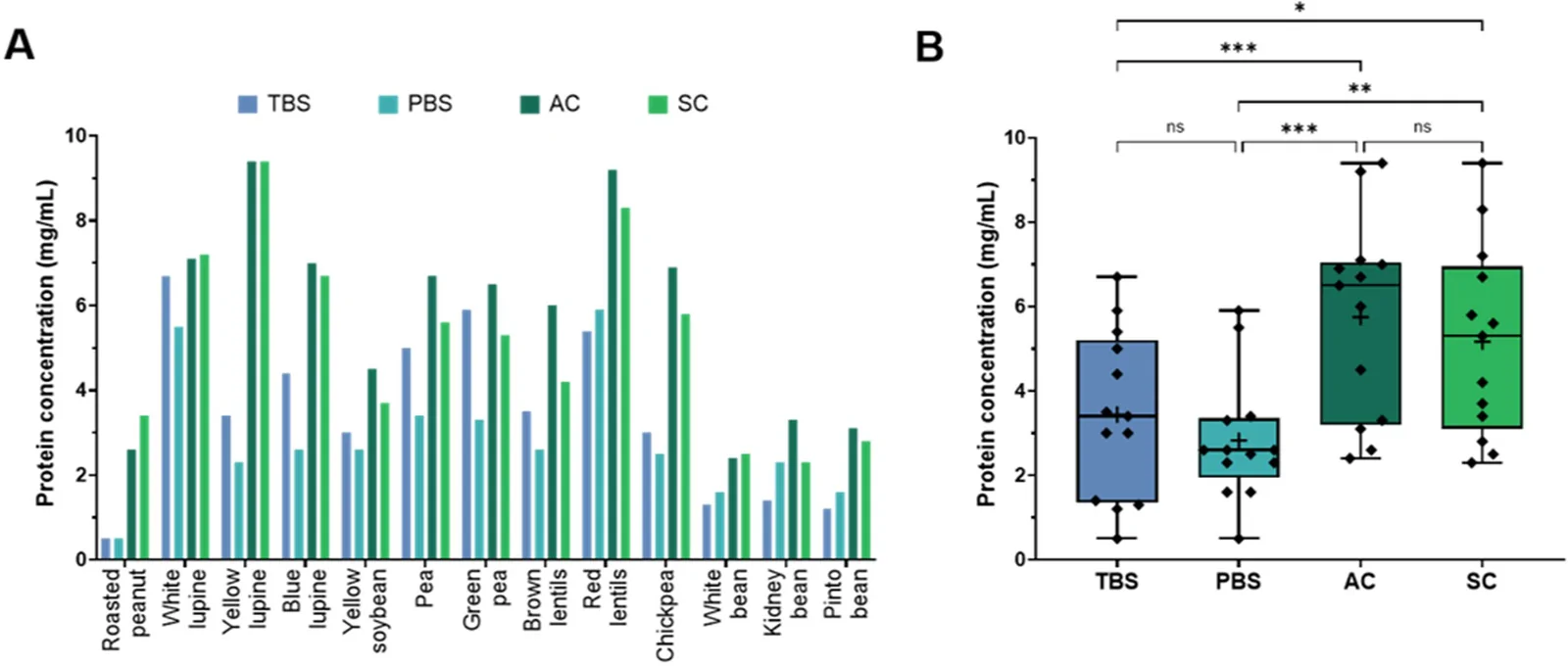
Impact of buffer on protein yield. **(A)** Individual protein concentration obtained from various legume seeds (Table 1), and **(B)** statistical comparison of protein concentration depending on different extraction buffers (TBS, PBS, AC, SC) as analysed by one-way ANOVA followed by the Tukey-test, ns = non-significant, * = *p* < 0.05, ** = *p* < 0.01, *** = *p* < 0.001.

The proteins present in each legume seed extract, using different extraction buffers, were profiled in 13% SDS-PAGE under reducing conditions (Figure 3). For the majority of studied legumes, the four extraction buffers demonstrated comparable protein profiles within each seed material (white and blue lupine, yellow soybeans, brown and red lentils and white beans, kidney and pinto beans), but with some variation in band intensities. Larger differences in protein patterns were observed in roasted peanuts, yellow lupine, peas and chickpeas.

**Figure 3:**
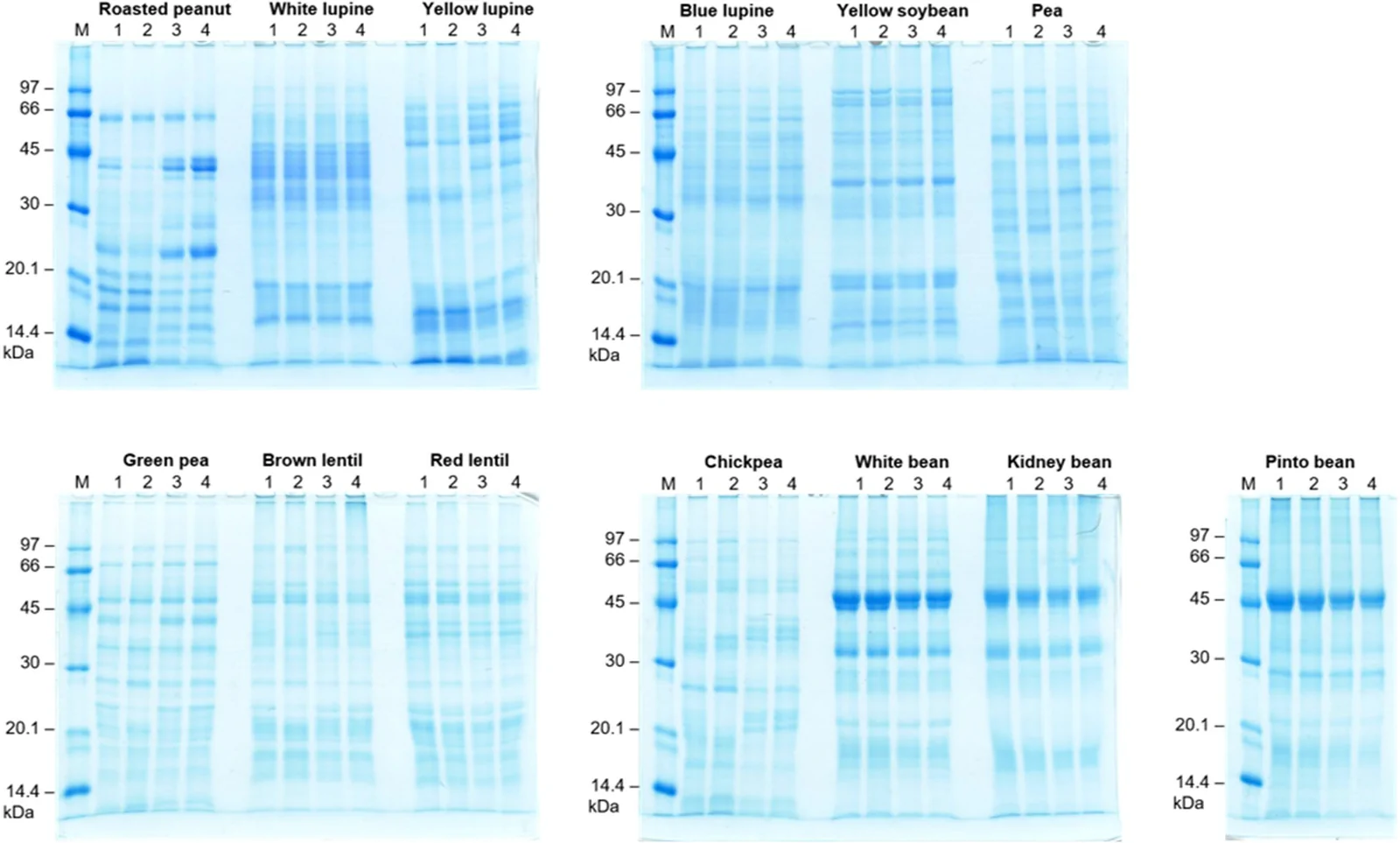
Protein profiles of extracts from in-house legume material (Table 1), according to coomassie-stained SDS-PAGE under reducing conditions, obtained using different extraction buffers. M = Marker, 1 = TBS, 2 = PBS, 3 = AC, 4 = SC.

Extractions made with AC and SC buffers did not differ much in the protein profiles; however, slight differences in band intensities were observed between the two buffers. This difference is principally observed in the roasted peanut extract, where SC enhances the extraction of the proteins in the range between 20.1–45 kDa. Most of the extracts prepared with TBS have profiles similar to those extracted with AC or SC buffers, but principal differences are observed in roasted peanut, yellow lupine, pea and chickpea, where some bands in the area between 20.1–45 kDa appear weaker than those extracted with the other two buffers. PBS buffer seems to have lower extraction capacity for proteins in the 20.1–45 kDa range, where the protein bands are weaker or absent. In contrast, extraction with TBS and PBS buffers enriched the proteins below 20.1 kDa and, in the case of the bean seeds, they also enriched proteins around 45 kDa. Together, the AC buffer was selected for further optimization steps, because it resulted in good extraction yields, balanced protein profile, and the possibility of complete removal of buffer components with a subsequent lyophilization step.

### 3.2 Refinement of extraction conditions for higher protein yield from ALLPreT study material

After selecting the AC buffer, the extraction conditions were further refined using selected ALLPreT legume seed material (Table 2), i.e., roasted peanut, soybean, winter pea and beluga lentil. First, a weight-to-buffer ratio of 1:5 (w/v) was compared to the initially applied 1:20 ratio to further increase yield of extracted protein, combined with extraction at room temperature for 1 h instead of initial extraction at 4 °C for 4 h. Protein concentration (Supplementary Table S3) and protein profiles (Supplementary Figure S1) under these conditions revealed that, with lower ratio and higher temperature at reduced extraction time, the amount of extracted protein increased while protein profiles did not change in general. Based on these results, a ratio of 1:5 and extraction with AC buffer at room temperature for 1 h were selected for subsequent experiments.

Lipophilic components may affect the solubilization of proteins with impact on extraction efficacy. Thus, the effect of defatting on extraction and protein composition was assessed. Seed powder from various ALLPreT legumes were defatted with n-hexane. This organic solvent was chosen for its low polarity and extensive use as a processing aid in the manufacture of foods. Defatted and non-defatted seed powder were extracted using AC buffer as described above. Protein concentrations and electrophoretic protein patterns between treatments were compared.

Protein quantification (Supplementary Table S4) showed a threefold increase in protein concentration for roasted peanut, with 14.4 mg/mL of protein obtained using the non-defatted seed powder against 45.9 mg/mL using the defatted one. On the other hand, unroasted peanut, sweet blue lupine and beluga lentils, presented similar extracted protein amounts in the non-defatted vs. defatted extracts. Soybean, chickpea and field bean showed somewhat higher protein content in the defatted extract, while it was lower in the defatted winter pea extract. However, the statistical analysis showed that, in general, there is no group-specific significant difference in the amount of protein extracted (Figure 4A) from non-defatted vs. defatted seed powder, although the threefold difference for roasted peanut was large. Protein profiles analysed by SDS-PAGE (Figure 4B) did not show remarkable differences in the protein profile or distribution of the proteins from each extract, although a slight variation in band intensities can be observed around 20.1–45 kDa in roasted and unroasted peanut extracts. Except for peanut, AC buffer was able to extract comparable amounts and patterns of legume proteins whether or not the seed powder is defatted.

**Figure 4:**
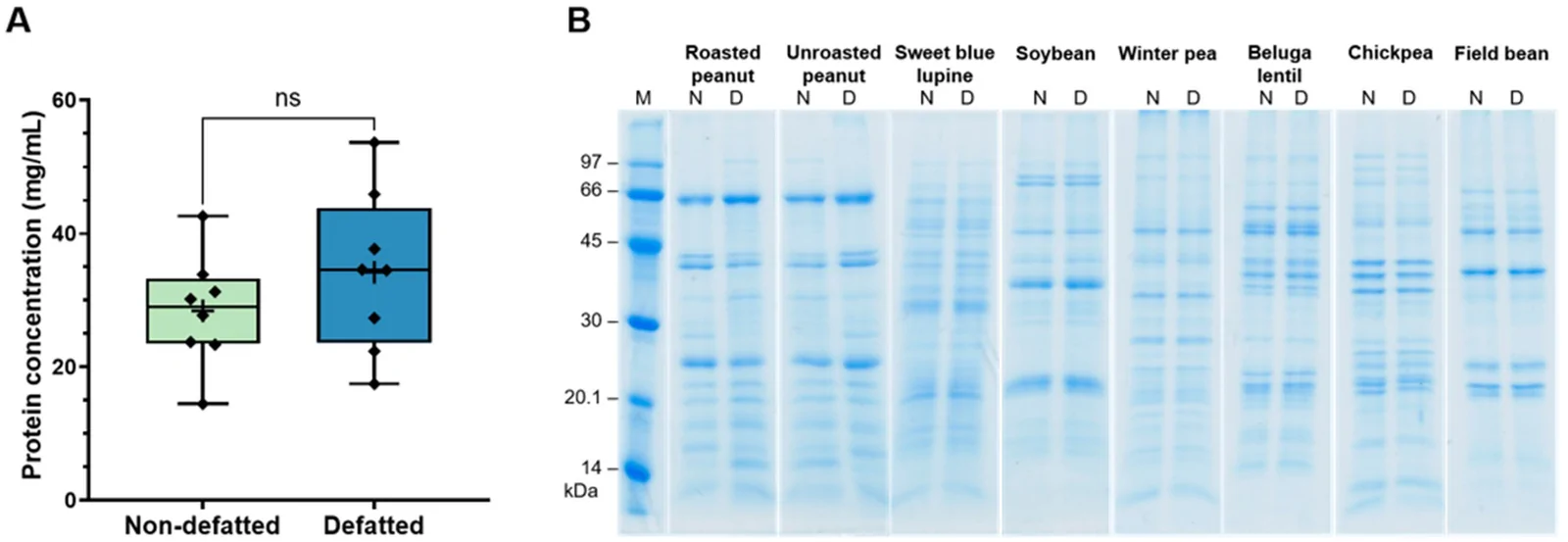
Effect of defatting on protein extraction from ALLPreT legume seed material (Table 2). **(A)** Comparison of protein concentration obtained after AC buffer extraction using non-defatted and defatted seed material. Statistical significance was assessed by paired Student's *t*-test (*p* < 0.05). ns = non-significant. **(B)** Comparison of protein profiles of legume protein extracts from N = non-defatted and D = defatted seed powder using AC buffer, as analysed by SDS-PAGE under reducing conditions. (M, molecular weight marker).

Because defatting of the roasted peanut seed material improved quantitative protein extraction yield, qualitative immunoblot analysis was done to investigate potential effects of defatting on protein immunodetection. Western blot analysis of both non-defatted (Figure 5A) and defatted extracts (Figure 5B) showed that, in general, the immunostaining of proteins using anti-peanut pAb and mAbs was weaker in the defatted extract in comparison to the non-defatted one. Besides a reduced signal intensity in the defatted extract, a change in the binding pattern of low molecular weight proteins (≤ 20.1 kDa) with rabbit anti-peanut IgG can be observed (lanes 2 and 3). In lanes 5 (anti-Ara h 1), 6 (anti-Ara h 2) and 7 (anti-Ara h 3) the band slightly above 14 kDa is clearly weaker in the defatted extract. The change in signal intensity suggests that, although treatment with n-hexane increases protein yield from roasted peanut, immunological properties of the protein in the extracts might be negatively affected by the solvent n-hexane. Nonetheless, we cannot conclude that the non-defatted extract is generally of lower quality, neither we observed any negative effects associated with the non-defatted extract. Thus, avoidance of a defatting step simplifies the protocol and eliminates the risk that potentially relevant lipophilic components might be removed during a defatting step.

**Figure 5:**
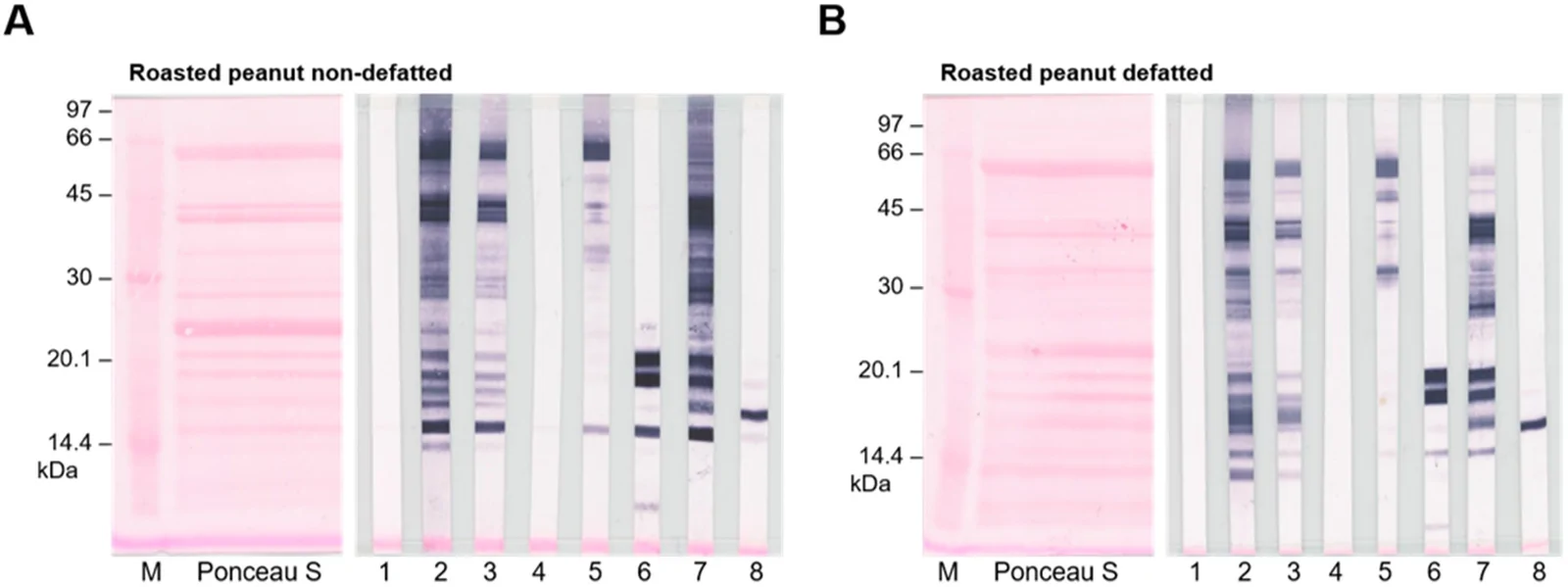
Assessment of defatting of ALLPreT peanut seeds (Table 2) on protein immunodetection. **(A)** non-defatted and **(B)** defatted roasted peanut extracts. M = Marker, Ponceau S = Ponceau S stain of total protein extract, 1 = buffer control (anti-rabbit IgG), 2 = rabbit pAb anti-peanut (1:10,000), 3 = rabbit pAb anti-peanut (1:60,000), 4 = buffer control (anti-mouse IgG), 5 = mAb anti-Ara h 1 (1:10,000), 6 = mAb anti-Ara h 2 (1:10,000), 7 = mAb anti-Ara h 3 (1:1,000), 8 = mAb anti-Ara h 6 (1:2,000).

### 3.3 Upscaling of the optimized extraction protocol retains quality of protein extracts

The optimized protocol [grinding under liquid nitrogen, non-defatted seed powder, AC buffer, 1 h extraction at room temperature, ratio 1.5 (w/v)] was upscaled for all ALLPreT legume seeds (Table 2) from 0.25 to 1 g in 5 mL during the optimization phase to 20 g in 100 mL to achieve higher amounts of extracted total protein to cover the needs of the project partners throughout the entire period of the project (years 2023–2026; range 1–5 g per protein extract). When the optimized extraction protocol was applied to the small-seeded legumes fenugreek, alfalfa and clovers, the protein extracts became highly viscous and difficult to pipette. Accordingly, in small-seeded legumes, the previous extraction ratio of 1:20 (w/v) between the weight of ground seeds and buffer volume was kept, which finally resulted in adequate fluidity of the extracts. The aim of the present study was to characterize the protein composition of our optimized legume extracts. If evaluation of the effect of extraction ratio on allergen composition is another goal for a harmonized protocol across legume species, such aspect would require further analysis. The performance of the final extraction protocol was assessed by quantifying protein concentration of the legume seed extracts, and the estimated extraction efficiency was then calculated against cited reference values as described in the “Methods” section. The detailed values and the reference protein contents used to estimate the extraction efficiency are listed in Supplementary Table S5. Although this might have limitations with regard to the true reference value of the underlying sample, the estimates suggested that the optimized protocol enables extraction of a relevant portion of the total seed protein (Supplementary Table S5).

Protein concentrations of common legumes (Figure 6A) obtained across the optimization steps were generally consistent upon up-scaling, compared to non-scaling (data not shown). Similarly, when the extraction protocol was applied to the small-seeded legumes with the adjusted ratio, the protein concentrations were somehow similar to the amounts observed for the common legumes from the ALLPreT study material during the optimization under equivalent dilution conditions (Supplementary Table S5). This indicates consistency of the protocol across seed material and scalability maintaining the same proportions on protein concentration.

**Figure 6:**
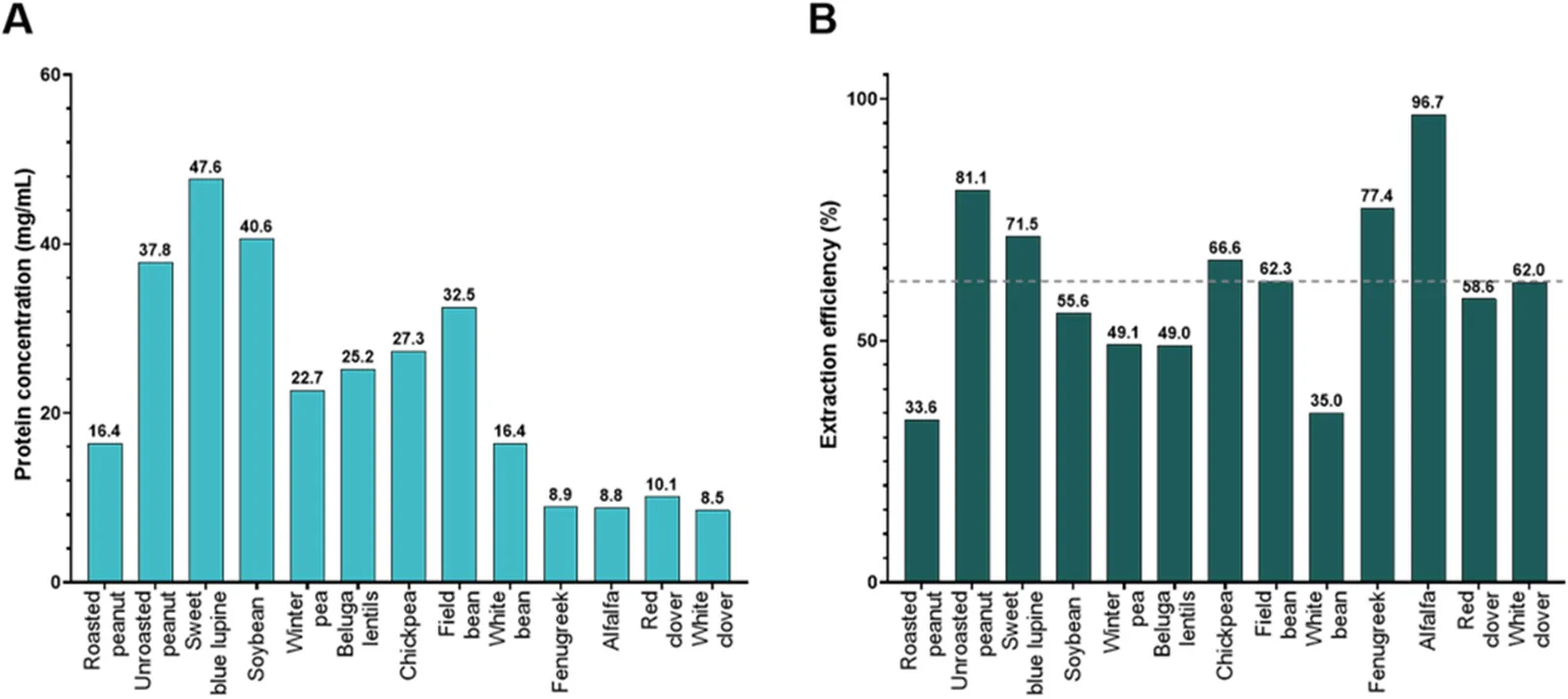
Assessment of protein yield from ALLPreT legume seed material (Table 2) by upscaling of the optimized protein extraction protocol. **(A)** Protein concentration obtained after extraction from each legume seed material. **(B)** Extraction efficiency calculated in relation to referenced protein content of the corresponding seed material. Extraction ratios are 1:5 (w/v) for common legumes and 1:20 (w/v) for small-seeded legumes. The dashed line represents the median of the values.

The final optimized protocol for the extraction of proteins from the various legume seed materials estimated extraction efficiencies with a median of 62.3% (range 33.6–96.7%) in relation to the reference protein content (Figure 6B). The highest estimated extraction efficiency (96.7%) was obtained from alfalfa, the lowest in white bean (35%) and roasted peanut (33.6%). Unroasted peanut presented an extraction efficiency above median of all investigated legumes (81.1%). Overall, the estimated extraction efficiencies were adequate for the majority of the investigated seed materials using the optimized protocol.

### 3.4 Proteomic profiling of ALLPreT legume seed extracts reveals the presence of relevant allergens

Mass spectrometry was applied to characterize the ALLPreT legume seed (Table 2) total protein extracts. Reported allergens in allergen databases, annotated homologous proteins, and identified proteins were classified under the corresponding protein families, based on a protein family naming framework as described in the Supplementary material (Supplementary methods), to assess the relative abundance of proteins potentially belonging to allergenic families across all legume seed extracts (Supplementary Tables S6–S8). The allergenic protein relative abundance in each extract differed among the legume seed extracts (Figure 7, Supplementary Table S9), with the highest abundance of identified allergens shown in the roasted peanut extract and the lowest in white bean extract. Major seed storage protein families such as 11S globulins (legumins) and 7S globulins (vicilins), were detected in all extracts, while 2S albumins were not found in winter pea, beluga lentil, field bean and white bean. However, the proteomic analysis revealed the presence of other albumins in the form of seed albumins in those extracts. Other minor allergenic proteins such as PR-10, profilins, non-specific lipid transfer (nsLTP), oleosins and defensins were found to a lesser extent. Although reported as an allergen for mung bean (*Vigna radiata*), cytokinin-specific binding protein (CSBP) was not found in our legume seed extracts. However, these findings demonstrate that the extraction protocol was able to extract proteins from relevant allergenic protein families from both common and small-seeded legumes.

**Figure 7:**
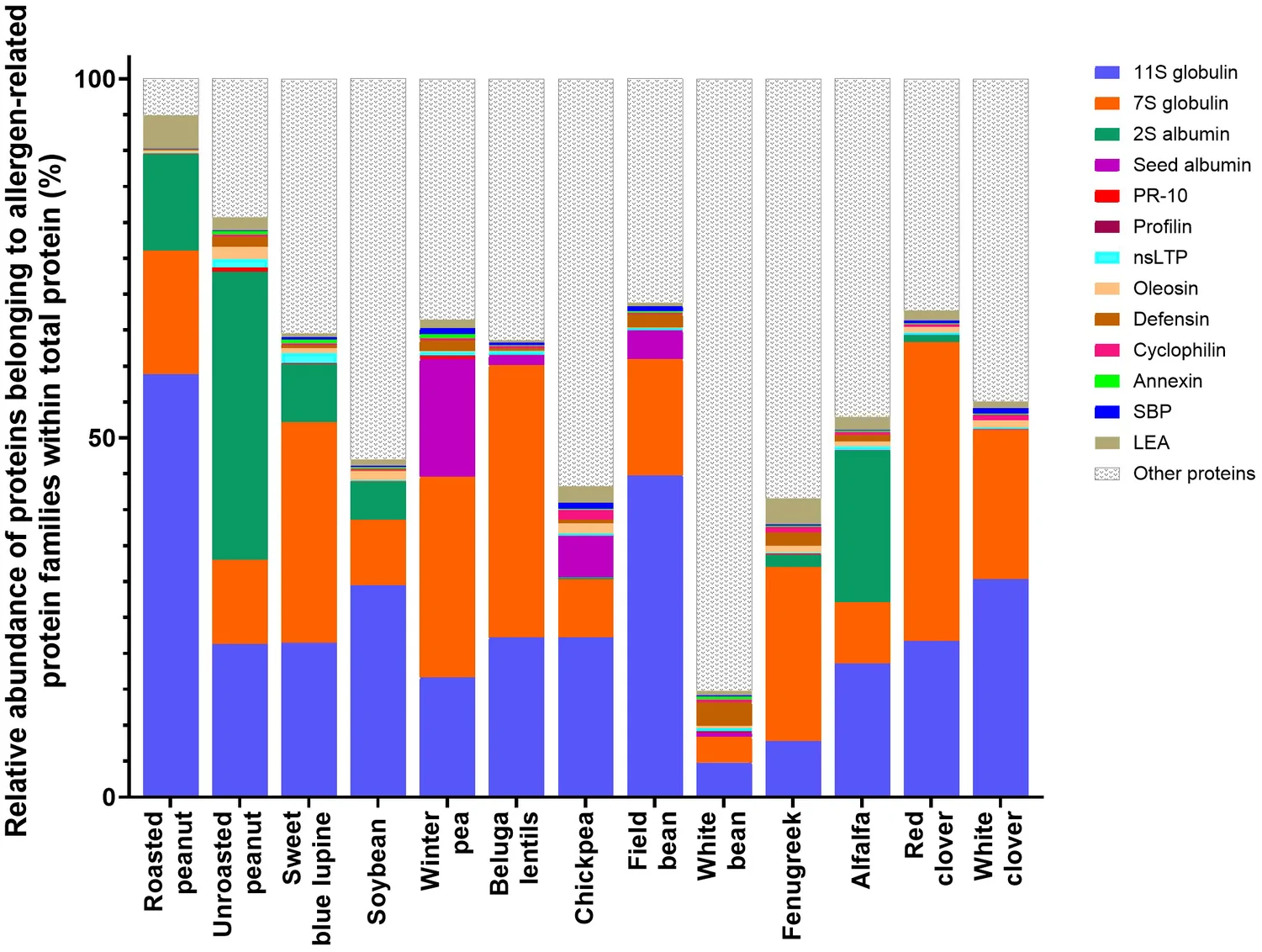
Relative abundance of proteins belonging to allergen-related protein families within the total protein in each ALLPreT legume seed (Table 2) extract, based on the protein family naming framework. Each legume seed extract was digested in triplicate, and each digest was injected in triplicate by liquid chromatography-tandem mass spectrometry (LC-MS/MS) analysis. Stacked bars indicate the abundance of the protein families. PR-10, pathogenesis-related 10 protein; nsLTP, non-specific lipid transfer protein; SBP, seed biotinylated protein; LEA, late embryogenesis abundant protein.

### 3.5 Optimized legume seed extracts retain structural protein characteristics

The protein composition of the final ALLPreT legume seed (Table 2) extracts obtained using the optimized protocol were further analysed by SDS-PAGE under reducing and non-reducing conditions to compare protein profiles obtained along optimization steps and to examine the contributions of disulfide bonds in the assembly and subunit organization of the proteins. The protein profile of common legumes under reducing conditions (Figure 8A) were consistent across the optimization steps (Figure 4B, non-defatted), with equivalent band distribution and no evidence of major protein loss was observed. Moreover, small-seeded legumes resulted in characteristic protein band patterns across a broad molecular weight range, which indicated that the extraction is not biased toward proteins with particularly high or low molecular weights.

**Figure 8:**
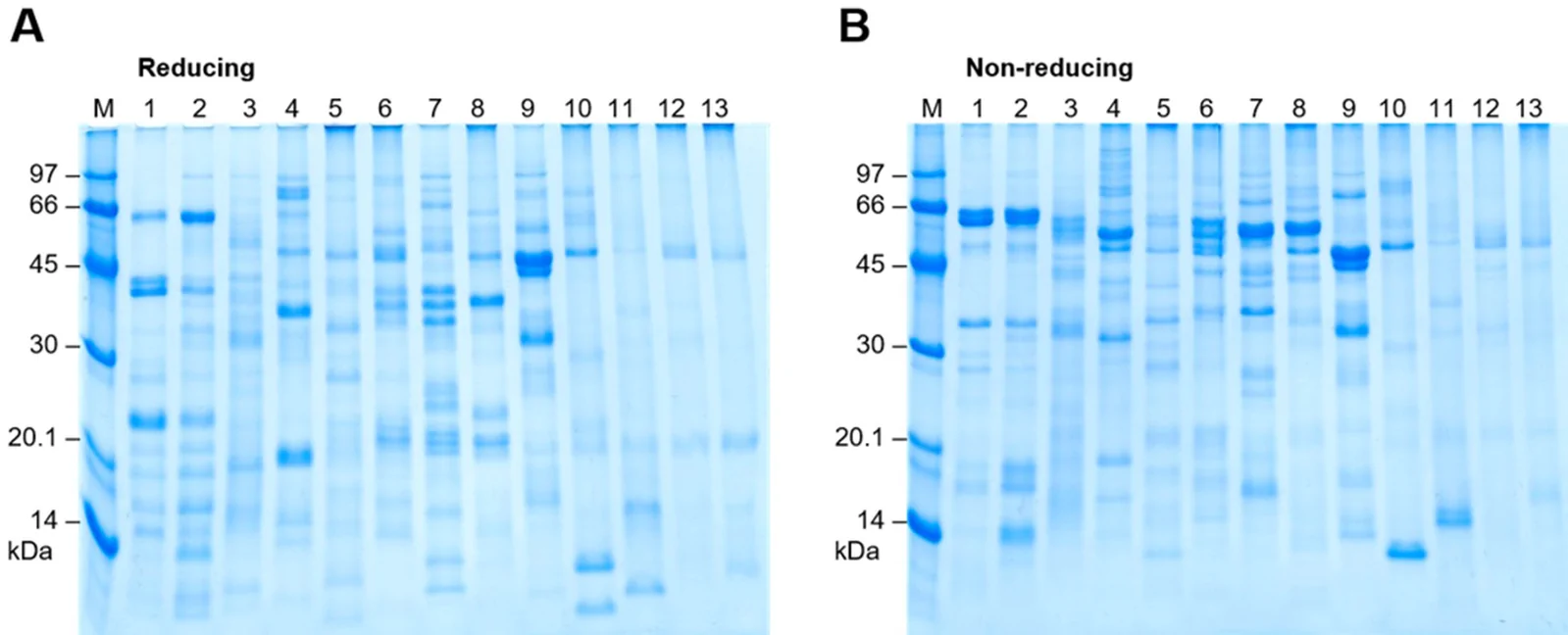
Protein profiles of ALLPreT legume seed (Table 2) extracts obtained with the final extraction protocol. Protein extracts were analysed by SDS-PAGE under **(A)** reducing and **(B)** non-reducing conditions to assess the impact of disulfide bonds on electrophoretic profile. M = marker, 1 = roasted peanut, 2 = unroasted peanut, 3 = sweet blue lupine, 4 = soybean, 5 = winter pea, 6 = beluga lentil, 7 = chickpea, 8 = field bean, 9 = white bean, 10 = fenugreek, 11 = alfalfa, 12 = red clover, 13 = white clover.

Comparison of protein profiles under reducing and non-reducing conditions revealed changes in protein patterns, consistent with the presence of disulfide-linked protein structures. While the gel under reducing conditions (Figure 8A) shows bands distributed along the gel and several bands under 14 kDa are observed, under non-reducing conditions (Figure 8B) the protein bands are predominantly present in the upper part of the gel and just a few protein bands under 14 kDa are observed. Together, these data suggest that structural protein characteristics, including disulfide bonds, are preserved.

### 3.6 IgG and IgE cross-reactivity of proteins in ALLPreT legume seed extracts

Immunodetection and antibody cross-binding of proteins in the ALLPreT legume seed (Table 2) extracts using the optimized extraction protocol were assessed by immunoblotting (Supplementary Figure S2) using IgG pAb against peanut (anti-peanut) and soybean (anti-soybean). When using these pAb, they were able to detect several proteins across the different legume seed extracts. However, patterns and intensity vary depending on the antibody tested. In general, IgG immunoblots demonstrated broad antibody recognition and cross-binding of proteins among legume species, indicating retained antibody-binding capacity and the presence of shared binding areas for detection.

The IgE reactivity of the extracts was later analysed in immunoblots using selected human sera (Supplementary Figure S3, Supplementary Table S1) from donors sensitized against legumes (Figure 9). First, three sera were selected out of 12 sera based on a broad IgE binding pattern against various relevant allergens from peanut, soybean, lentil and pea, and total protein extracts from lupine, pea, lentil, chickpea and fenugreek (Supplementary Figure S3, Supplementary Table S1). These IgE binding patterns were obtained from analysis with the ALEX^2^ array.

**Figure 9:**
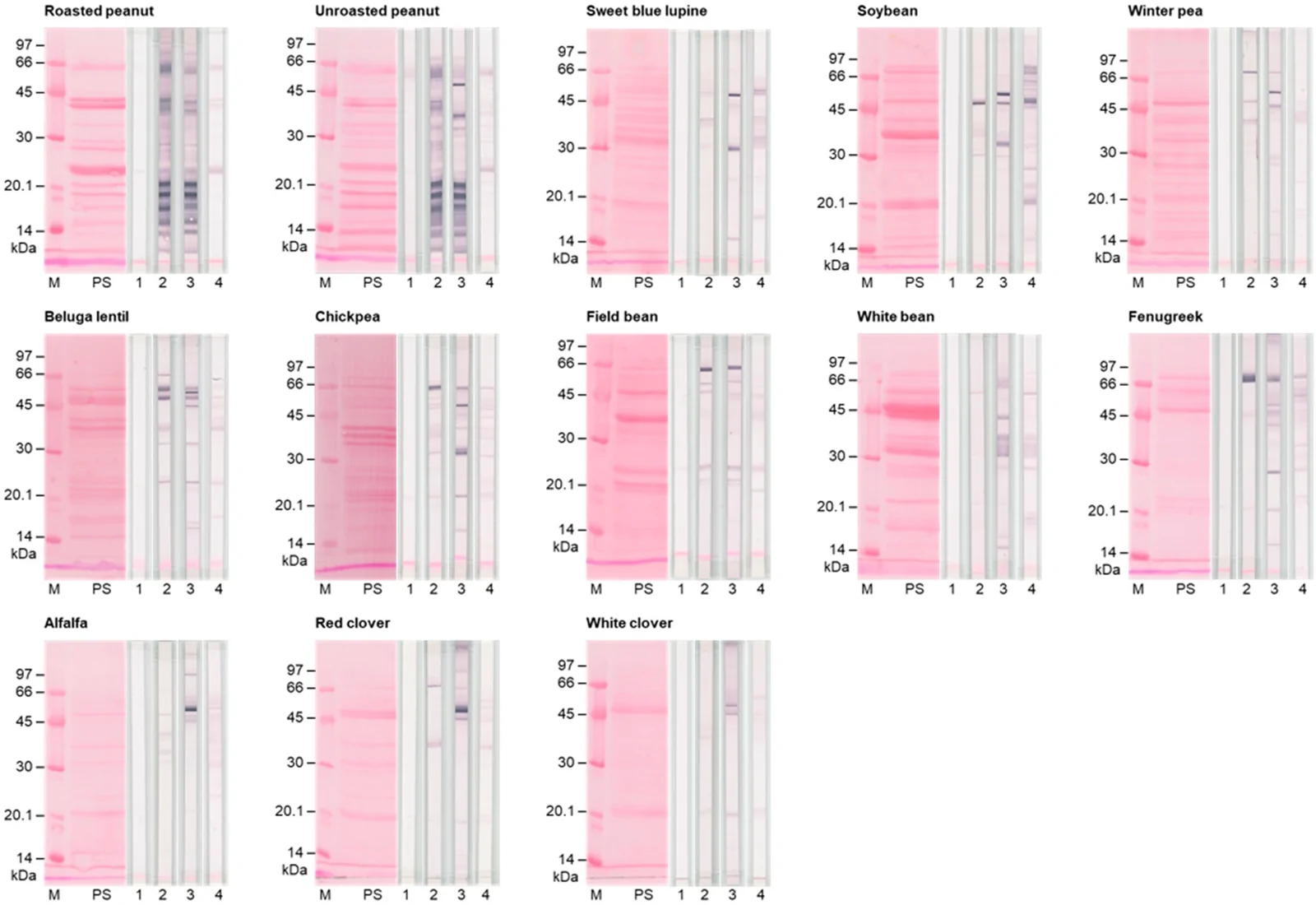
IgE immunoblot analysis of ALLPreT legume seed (Table 2) total protein extracts using selected human sera. M = marker, PS = Ponceau S staining of whole extract, 1 = buffer control (anti-human IgE), 2= PEI 7, 3 = PEI 11, 4 = PEI 1. All human sera were applied at a dilution of 1:20.

The selected sera PEI 7 and PEI 11 especially present elevated sIgE levels to peanut storage proteins (Ara h 1, Ara h 2 1, Ara h 2, Ara h 3, Ara h 6), profilin (Ara h 5) and PR-10 (Ara h 8), and to homologous allergens in soybean (Gly m 5, Gly m 6), lentil (Len c 1) and pea (Pis s 1), while PEI 1 shows prominent IgE binding to storage proteins in soybean (Gly m 5, Gly m 6) and peanut (Ara h 1, Ara h 3), and LTP-sensitization to peanut (Ara h 9) and lentil (Len c 3). All selected sera present IgE binding to total protein extracts of lupin, pea, lentil, chickpea and fenugreek (Supplementary Figure S3, Supplementary Table S1).

Differences in IgE-detection patterns (Figure 9) were observed when using the selected human sera. In general, IgE recognition patterns differed among the legume seed extracts for the three sera tested with only limited number of protein bands detected consistently across sera, highlighting different IgE-binding areas in the proteins. Notably, the three sera tested also recognized some proteins from the small-seeded legume extracts, and serum PEI 11 (lane 3) seems to detect strongly the protein bands around 45–66 kDa. Together, these findings confirm that the extracted proteins are recognized by human IgE and support the suitability of the extracts for further downstream analyses.

### 3.7 Functional IgE-crosslinking activity of legume seed protein extracts in MRA

The functional IgE reactivity of the extracts was evaluated using a humanized mediator release assay with passively sensitized RBL-2H3 cells. The human sera PEI 1, PEI 7 and PEI 11 were used to study mediator release to enable comparison between IgE recognition and functional effector cell responses. MRA curves of the three sera are displayed in Figure 10. All depicted release-data have been corrected for potential unspecific release due to cytotoxicity that may result from the intrinsic properties of the sera, or legume extracts, related endotoxin levels (Supplementary Table S10) or lectin levels (Supplementary Table S9). For identification of such unspecific effects, serum and antigen controls were run. The serum controls, in the absence of antigen, showed no unspecific release, with release values between 4.4% and 7.8% of total release, which can be considered background. The antigen controls were run in triplicate for each of the three highest protein concentrations (30 µg/well, 2.5 µg/well, 0.208 µg/well) of each legume extract and in the absence of serum. In those wells with elevated unspecific mediator release, we visually observed cell death at varying levels. For the 0.208 µg/well antigen control, mediator release was below 5% of total release in all legume extracts, which can be considered background. Likewise, for the 2.5 µg/well control, in all legume extracts, except for white bean, release was between 3.0% and 6.3% of total release, which can be considered background. In white bean, release was 39.4% of total release. The calculated endotoxin levels of all negative legume extracts ranged between 0.03 and 8.8 EU/mL at this antigen concentration, while it was 0.17 EU/mL in white bean. We conclude, that the endotoxin levels at this protein concentration do not appear to cause unspecific mediator release. With 11.6% of total protein, white bean presented the highest level of lectin in all legume extracts, which ranged between < 0.1% and 1.2% for all legumes except for white bean (11.6%) and Beluga lentil (6.9%). Taken together, we consider the high lectin concentration as a possible explanation for the non-specific mediator release by white bean extract at the second highest protein concentration investigated. At the 30 µg/well antigen control, several legume extracts (soybean, Beluga lentil, winter pea, field bean, white bean, fenugreek, alfalfa, white clover) led to unspecific release at or above 10% of total release (range 10.5%—83.2%). Except for soybean (0.4 EU/mL), all legume extracts with unspecific release had calculated endotoxin levels above 1 EU/mL (range 1.3 EU/mL—105.3 EU/mL) at this antigen concentration. This might suggest, at least to a certain extent, causal relation to the unspecific release in several of these legume extracts by potential activation through Toll-like receptor 4. In all other legume extracts (peanut roasted, peanut unroasted, blue lupine, chickpea), with background-like mediator release between 4.1%—5.8% of total release, calculated endotoxin levels were at or below 1 EU/mL. However, red clover extract, with calculated 95.4 EU/mL endotoxin at this antigen concentration, also presented background-like release of 4.4% of total release.

**Figure 10:**
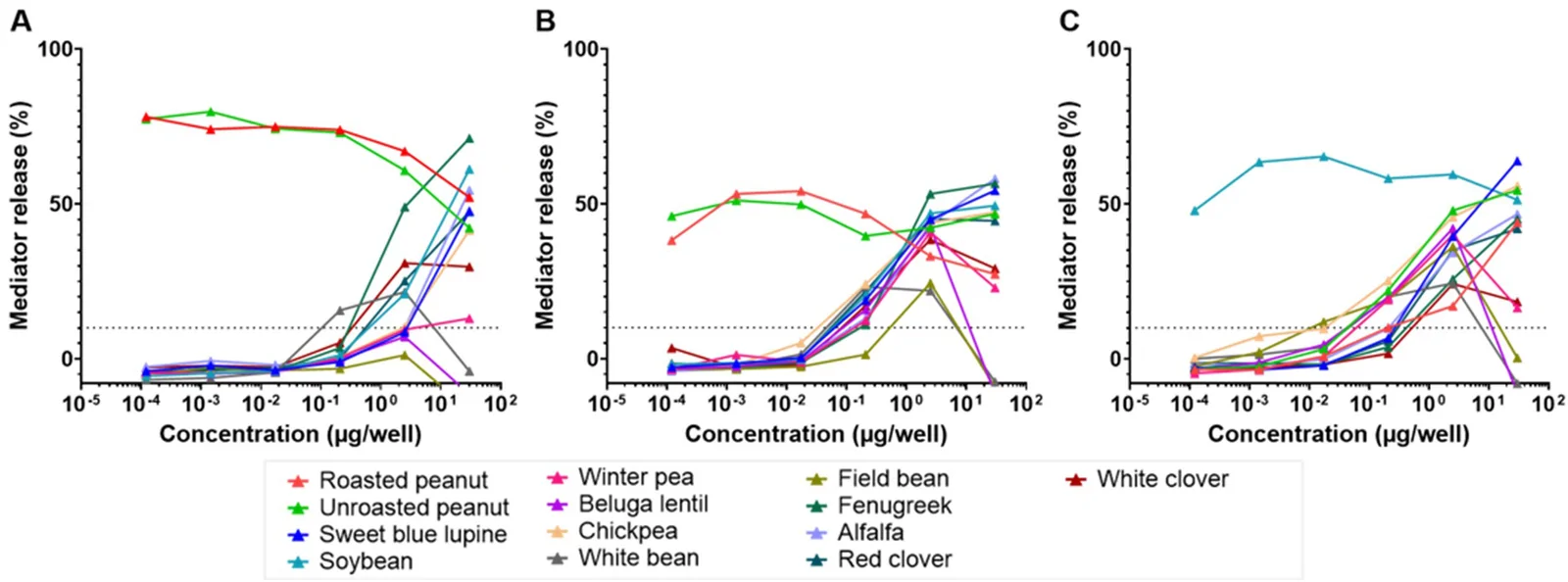
Assessment of capacity of ALLPreT legume seed (Table 2) extracts to induce effector cell activation using RBL-2H3 mediator release assay. Mediator release is expressed as a percentage of total release after passive sensitization with human serum IgE and stimulation with a concentration series of legume seed extracts (10^−4^ to 3*10^1^ µg/well). **(A)** PEI 7, **(B)** PEI 11 and **(C)** PEI 1. The dotted line indicates the cut-off (mean + 2*SD of sensitized but non-stimulated cells, *n* = 8), responses above the cut-off were considered positive.

In summary, for the highest protein concentration investigated, unspecific mediator release was observed in several legumes, likely related to elevated levels of lectin and/or endotoxin. Except for white bean, all protein levels at or below 2.5 µg/well were non-cytotoxic for all extracts. For white bean all levels below 2.5 µg/well were non-cytotoxic. After correction against observed unspecific release, the MRA curves of the three sera (Figure 10) display protein-specific mediator release.

Mediator release varied among the sera and the legume seed extracts tested. Mediator release was observed in sera with elevated sIgE levels. For instance, PEI 7 and 11 had high *β*-hexosaminidase release with both peanut extracts (roasted and unroasted) and high sIgE levels for peanut allergens, while PEI 1 had higher release with the soybean extract. This is in line with the immunoblot results and demonstrated that differences in IgE recognition (Supplementary Figure S3, Supplementary Table S1) may translate into differing cellular activation. In addition, extracts with only a few bands recognized, generally presented low (above the cut-off) or no mediator release (below the cut-off). The sera also showed mediator release with the small-seeded legume extracts, which was comparable to the common legumes.

## 4 Discussion

The method of protein extraction plays a critical role in the detection, quantification and immunogenicity of proteins (20, 30). While the use of additives like reducing agents (e.g., dithiothreitol), detergents (e.g., sodium dodecyl sulphate and Tween 20), complexing (e.g., EDTA) and denaturing (e.g., urea/thiourea) agents can enhance protein extraction and thus, enhance the analytical sensitivity in antibody binding assays such as ELISA and Western blot (21, 31, 32), their use may not be compatible with biological *in vivo* and *in vitro* models of allergenicity, as they can affect cell viability, alter physiological responses or do not reflect biological conditions. Furthermore, extraction parameters including buffer composition, pH and sample processing directly influence protein solubility and immunoreactivity, which might affect outcomes in allergenicity assessment (33, 34). Consequently, variability in extraction methodologies represents a significant source of inconsistency across studies. In addition to this, the lack of harmonized protocols and reference materials in current risk assessment limits the comparability of data generated across different experimental methods (18). In this work, an optimized protein extraction protocol was established for the extraction of proteins from various legume sources, with consistent extraction yields and protein distribution across sources, to generate material suitable for its use on both *in vitro* cellular (35) and *in vivo* methods (36), to be applied within the ALLPreT consortium. The buffer and parameters for protein extraction were selected based on: 1) extraction performance (protein yield and profile), 2) suitability for *in vitro* and *in vivo* testing, 3) compatibility with lyophilization for subsequent distribution and long-term storage, and 4) preservation of immunoreactive properties of proteins even after lyophilization and reconstitution in water.

Comparison of extraction yields obtained using four different aqueous buffers without any denaturing additive demonstrated that the AC buffer (pH 9.0) provided significantly higher protein yields than those with lower pH such as TBS (pH 7.4) and PBS (pH 7.1). While yields were comparable to the SC buffer (pH 9.6), the protein profile obtained with the AC buffer showed a more consistent distribution across the diverse legumes tested. These findings align with established literature regarding the relationship between alkaline conditions and increased protein solubility (23, 30, 37). Additionally, SDS-PAGE and mass spectrometric analysis of AC-based extracts indicated that the extraction with AC buffer resulted in the presence of the most important allergenic proteins. This highlights the suitability of AC buffer to generate harmonized study materials where representative allergenic protein composition is fundamental.

After the selection of AC as a suitable buffer, extraction parameters such as sample-to-buffer ratio, incubation time, and temperature were adjusted ensuring higher protein yield while providing comparable protein pattern at reduced extraction time. No marked variations in the protein patterns were observed, indicating that the protocol is robust to minor procedural changes. In contrast, the investigation of a defatting step had a more pronounced impact on protein solubilization and composition. Although defatting of the seed material prior to extraction may increase the protein extractability, especially for roasted peanuts, it was observed that AC buffer alone was able to extract similar protein amounts in most cases. Besides that, defatting showed a negative effect on the immunochemical properties of the proteins, as shown in the case of roasted peanut extract, possibly due to structural changes that may alter conformational epitopes. Similar effects were reported by L'Hocine & Pitre (20), where defatting with hexane enhanced protein extractability but was also associated with variations in protein immunoreactivity. Furthermore, a primary goal of this work was to produce ALLPreT study material that preserves the native composition and structure of seed proteins, including lipid-associated components of the legumes. Ideally maintaining the complete matrix of the source is essential for accurate allergenicity assessment, where the interaction between proteins and their native lipid environment may influence their immunochemical properties and stabilize against gastrointestinal digestion (38, 39). Conventional defatting procedures frequently exclude lipophilic proteins, such as oleosins, which have been recognized as clinically relevant allergens (40). Although extraction and filtration inevitably alter the native composition of the source, retaining the natural oil body fraction within the extract, as shown by mass spectrometric analysis (Figure 7), ensures that the resulting material provides a broad allergenic profile, including minor allergens such as oleosins.

For the common legumes (peanut, soybean, sweet blue lupine, winter pea, beluga lentil, chickpea, white bean, field bean and fenugreek) the extraction performance was consistent during the optimization steps. No difference was observed after up-scaling the extraction. While, in case of the small-seeded legumes (alfalfa, fenugreek, red and white clover), the need for increased extraction volume is likely associated with their reported high galactomannan content (41), which markedly increases viscosity and limits effective mixing at lower buffer ratios. Nonetheless, the extraction performance appeared comparable to that observed for common legumes. By adjusting the extraction ratio, the AC buffer can be adapted to legume matrices with varying viscosities.

Proteomic profiling and SDS-PAGE demonstrated that the optimized extraction protocol recovered a broad range of proteins, including protein families that contain reported legume allergens. This is relevant for the intended use of the extracts as comparative study materials within the ALLPreT project and beyond, as it indicates that the extracts retain proteins with potential immunological relevance.

Legume seeds contain several classes of proteins, predominantly seed storage proteins such as 11S globulins (legumins), 7S globulins (vicilins) and 2S albumins (Figure 7). Structurally, 11S globulins (ca. 300–360 kDa) are composed of acidic (30–40 kDa) and basic (20 kDa) subunits that are linked by disulfide bonds (ca. 50–60 kDa), and which assemble as homo- or hetero hexameric proteins by non-covalent interactions. The 7S globulins (150–190 kDa) are homo- or hetero trimeric proteins composed of 45–60 kDa subunits bound together non-covalently. 2S albumins (11–18 kDa) consist of a small (3–5 kDa) and large chain (8–10 kDa, respectively) linked by disulfide bonds, except for peanut, where 2S albumins consist of a single peptide chain stabilized by intramolecular disulfide bonds (42, 43). Comparison of protein profiles in SDS-PAGE under reducing and non-reducing conditions suggest that the optimized extraction protocol preserves structural protein characteristics, including disulfide bonds.

For legumes with limited protein annotation in both allergen and protein databases, the application of the protein naming framework used for the identification of proteins belonging to conserved protein families, provided a more comprehensive basis for comparison among the various legume sources, compared to when only allergens present in the allergen databases were considered. However, protein assignment to allergen-related protein families was based on protein annotations and are potential putative allergens and should not be interpreted as confirmation of allergenicity. Thus, the reported abundances represent abundance of proteins belonging to allergen-related protein families rather than allergen abundance. A limitation of the present approach is that the framework relies on currently recognised allergens and allergen-related protein families. As genome sequencing and recombinant protein technologies advance, deeper *de novo* identification of protein sequences may support the identification by MS methods. Remarkably, among the major seed storage proteins, we observed differences in their relative abundances. We found higher levels of 11S globulins in the extract of roasted peanuts, while unroasted peanuts contained nearly half that amount; in contrast, unroasted peanuts contained nearly four times as much 2S albumins as roasted peanuts. This indicates that protein abundance can vary considerably depending on the source material and/or processing such as roasting. Moreover, no 2S albumins were found in winter pea, beluga lentil, field bean and white bean.

Casale et al. (44) investigated the product-to-product variability in Ara h 1, 2, 3, 6, and 8 levels in peanut-containing foods, finding that protein content varied significantly among different peanut kernels, with a difference of up to 15-fold for Ara h 3 (11S globulin) and a threefold difference in the levels of Ara h 2 and 6 (2S albumin). Similarly, studies focusing on allergen content in different soybean varieties demonstrated that location of cultivation and genetics affected allergen quantitation (45, 46). These studies underline that biological variation in allergen content between cultivars, their location of cultivation, and the products made thereof exist and have to be considered.

A particular challenge in analysing the data was the lack of proteomic literature on some of the common and small-seeded legumes included in the ALLPreT material. While the protein profiles of the common legumes have been widely studied (47–51) and thus, allow the comparison of the allergen profiles obtained with our optimized protocol, there are only a few or no reference data available from alfalfa, red and white clover seeds, which makes it difficult to confirm or compare protein profiles. Moreover, the available literature on these small-seeded legumes mainly focused on leaves, sprouts or other plant tissues rather than the seeds. However, based on the extraction performance observed with the common legumes that remained reproducible across the different legumes and the proteomic data, our findings provide baseline data for those species.

Besides the performance on protein extraction, AC buffer was selected to ensure compatibility with the lyophilization, a critical step for the distribution and long-term storage of the reference material. Commonly used buffers for protein extraction such as PBS and TBS contain considerable amounts of salts which often concentrate during the freeze-drying step. This can lead to extreme pH variations and high ionic strength which can potentially induce protein aggregation or degradation (52, 53). In contrast, ammonium carbonate is an unstable salt that decomposes into gaseous ammonia, carbon dioxide and water upon freeze-drying (54). Thereafter, no buffer components remain, and the resulting freeze-dried protein extracts can be resolved in any other buffer, which makes the extracts principally compatible with any conditions required in downstream *in vitro* and *in vivo* assays. The volatility of the AC buffer is a core highlight. However, drastic pH changes during lyophilization may still cause irreversible denaturation of sensitive proteins. If there were any indications of this, it should be addressed in future research.

The suitability of the harmonized legume seed extracts was validated by confirming their immunological and functional reactivity. The initial screening with polyclonal anti-peanut and anti-soybean IgG antibodies confirmed that after the extraction process the proteins retained their immunoreactivity. Additionally, immunodetection using human sera from sensitized individuals demonstrated that IgE-binding capacity was maintained. Interestingly, not only the proteins from the common legumes (peanut, soybean, other) to which the serum donors were sensitized were recognised, but also the small-seeded legumes alfalfa and clovers were able to bind to IgE from these serum donors (Figure 9, Supplementary Table S1). This demonstrates some degree of IgG and IgE cross-binding between the common and small-seeded legumes, which are potential examples of novel foods. Furthermore, the extracts demonstrated functional IgE cross-linking capacity of basophils using humanized RBL cells. Our primary aim was to investigate the quality of the optimized legume extracts with regard to future use in allergenicity assessment studies. Demonstration of IgE-binding and IgE-crosslinking characteristics are relevant quality criteria before using such material. It must be emphasized that the MRA data do not allow for any conclusions regarding clinical relevance, since the serum donors were not sufficiently characterized clinically, and this was never an objective. However, the demonstrated IgE-crosslinking properties qualify the legume extracts for further use in *in vitro* allergenicity assessment studies with clinically well-characterized subjects as a next step. Notably, some extracts caused cytotoxicity when they were used in the highest concentration (30 µg/well). Except for white bean, all other legume extracts did not show cytotoxic effects at protein levels of 2.5 µg/well and below. For white bean, all levels below 2.5 µg/well were non-cytotoxic. Notably, white bean and Beluga lentil had the highest levels of lectins (Supplementary Table S9), which have been widely reported for cell toxicity at high concentration (55). Moreover, elevated endotoxin levels (Supplementary Table S10) might contribute to cytotoxicity observed for other legumes at the highest protein level investigated. The displayed mediator release data (Figure 10) are corrected for unspecific release due to sera or antigen. In summary, all investigated legume extracts presented IgE-crosslinking properties. Overall, the results indicate that the optimized extracts retain functional immunological activity (IgE-crosslinking) and, at adequate concentrations, are suitable for use in cell-based immunoassays. All together, these results establish the produced extracts as well characterized comparative study material for use in the establishment of new models in the allergenicity assessment of novel foods within the ALLPreT consortium. Once their suitability has been demonstrated in various immunochemical and cellular *in vitro* assays as well as in *in vivo* animal models, this protocol for the preparation and characterization of legume protein extracts may be proposed for future allergenicity studies of novel legume foods.

## Data Availability

The datasets presented in this study can be found in online repositories. The names of the repository/repositories and accession number(s) can be found below: http://www.proteomexchange.org/, PXD079632.
