# Supplementary material for: The ALLPreT panel of legume allergen preparations: characterization of optimized protein extracts for allergen composition, IgE-binding and IgE-crosslinking properties
Source: Front Allergy. 2026 Sep 28;7:1938923. doi: 10.3389/falgy.2026.1938923 (PMC13654845; doi:10.3389/falgy.2026.1938923)
Supplement: Supplementary file 1 [file Supplementaryfile1.pdf]

Supplementary Material

1 Supplementary Methods

1.1 Relative abundance of proteins by LC-MS/MS

1.1.1 Sample preparation

1.1.1.1 Reduction and alkylation

Lyophilized legume extracts were resuspended at 5 mg/mL in PBS (137 mM NaCl, 3 mM KCl, 8 mM Na_2HPO_4, 1 mM KH_2PO_4, pH 7.2). In triplicates, 2 µL of extract corresponding to 10 µg of protein per sample were transferred into LoBind tubes, and 14 µL of 50 mM Tris/HCl pH 8.8 buffer was added. Samples were reduced with 100 mM of tris (2-carboxyethyl) phosphine (TCEP) and alkylated with 2 µL of 400 mM chloroacetamide (CAA), bringing the final volume to 20 µL. Mixtures were incubated at room temperature for 20 minutes, heated in a dry heating block at 95°C for 5 minutes, briefly vortexed, placed on ice, and sonicated continuously in a BioRupter Pico sonicating water bath (Diagenode, Denville, NJ, US) on a 60-second on / 30-second off mode for 5 cycles at 8°C.

1.1.1.2 Sample digestion

Samples of 20 µL containing 10 µg of protein were diluted 3-fold by the addition of 40 µL of digestion buffer (10% acetonitrile [ACN] in 50 mM HEPES pH 8.5) and 200 ng of LysC (Fujifilm WAKO Pure Chemical Corporation, Osaka, Japan) was added at an enzyme:protein concentration ratio of 1:50). The mixture was vortexed and shaken in a Thermomixer comfort (Eppendorf SE) at 750 rpm at 37°C for 4 hours. The sample was then diluted a further ten-fold by adding 140 µL digestion buffer and 100 ng Trypsin (Thermo Scientific™) was added at an enzyme:protein concentration ratio of 1:100. The mixture was vortexed and shaken at 750 rpm at 37°C overnight. Trypsin was then inactivated with 200 µL of 2% trifluoroacetic acid (TFA) to obtain a final concentration of 1% TFA, vortexed, and centrifuged at 21,000 *g* for 10 minutes at room temperature to obtain the supernatant, which contains digested peptides.

1.1.1.3 Sample cleanup

The SOLAµ™ Solid Phase Extraction Hydrophobic Reversed Phase 2 mg/1 mL 96-well plate (Thermo Scientific™) columns were activated with 200 µL 100% methanol and centrifuged (SL 16 centrifuge, Thermo Scientific™) at 1500 g for 1 minute at room temperature. Centrifugation with the same conditions was performed after each subsequent step below. Columns were conditioned with 200 µL Buffer B (80% ACN, 0.1% formic acid [FA]) and twice with 200 µL Buffer A' (3% ACN, 1% TFA). Digested samples (400 µL) were loaded and centrifuged to retain peptides, followed by two washes of 200 µL Buffer A (0.1% FA). Peptides were eluted twice with 40 µL Buffer B' (40% ACN, 0.1% FA), supernatants transferred into clean 1.5 mL LoBind tubes and dried in a Speedvac at 60°C for 90 minutes. Dried samples were resuspended in 12 µL Buffer A* (2% ACN, 1% TFA), vortexed, sonicated for 4 minutes in a VWR Ultrasonic Cleaner USC300T (VWR International), and centrifuged at 18,000 g for ten minutes at room temperature. Peptide concentration was measured by loading 1 µL onto a DS-11 FX+ Series Nanodrop Spectrophotometer (DeNovix, Wilmington, DE,

US) and analyzed at A280 to obtain the peptide concentration. Samples were frozen at -20°C until liquid chromatography-tandem mass spectrometry (LC-MS/MS injection).

1.1.2 LC-MS/MS Analysis

In triplicates, peptides (500 ng protein per sample) were loaded onto a 2 cm C18 trap column (Thermo Scientific™ 164946), connected in-line to a 15 cm C18 reverse-phase EasySpray ES904 analytical column (Thermo Scientific™) using 100% of Buffer A (0.1% FA) at 750 bar, using the EASY-nLC™ 1200 LC system (Thermo Scientific™), and the column oven operating at 30 °C. Peptides were eluted over a 70 min gradient ranging from 10% to 60% of Buffer B (80% ACN, 0.1% FA) at 250 nL/min, and the Orbitrap Exploris™ 480 mass spectrometer (Thermo Scientific™) was run in data-independent acquisition (DIA) mode with field asymmetric ion mobility spectrometry (FAIMS) Pro™ Interface (Thermo Scientific™) with compensation voltage of -45 V. Full MS spectra were collected at a resolution of 120,000, with an automated gain control (AGC) target of 300% or maximum injection time set to 'auto' and a scan range of 400–1000 m/z. The MS2 spectra were obtained in DIA mode in the Orbitrap operating at a resolution of 60,000, with an AGC target 1000% or maximum injection time set to 'auto', a normalized HCD collision energy of 32. The isolation window was set to 6 m/z with a 1 m/z overlap and window placement on. Each DIA experiment covered a range of 200 m/z, resulting in three DIA experiments (400-600 m/z, 600-800 m/z and 800-1000 m/z). Between the DIA experiments, a full MS scan was performed for MS1 quantification.

1.1.2.1 MS data processing

MS data processing is based on nine individual MS data sets for each legume, which originate from triplicate digesta of each legume extract (1.1.1), and triplicate injection per digesta (1.1.2). Raw data were processed with Spectronaut® 20 (Biognosys), and the spectra were searched against the following taxonomies at the species level unless otherwise stated: peanut *Arachis hypogaea* (ID 3818: 102,020 entries), soybean *Glycine max* (ID 3847: 85,130 entries), pea *Pisum sativum* (ID 3888: 66,517 entries), chickpea *Cicer arietinum* (ID 3827: 31,239 entries), white bean *Phaseolus vulgaris* (ID 3885: 32,796 entries), sweet blue lupine *Lupinus angustifolius* (ID 3871: 31,389 entries), lentil Fabeae family (ID 163743: 107,132 entries), field bean *Vicia faba* (ID 3906: 36,660 entries), fenugreek Trifolieae tribe (ID 163742: 311,473 entries), alfalfa *Medicago* genus (ID 3879: 94,640 entries), red clover *Trifolium pratense* (ID 57,577: 60,478 entries), and white clover *Trifolium* genus (ID 3899: 215,542 entries). The FASTA database files for the above taxonomies were assessed in UniProt on 8 January 2026. The following parameters for protein identification searches were adopted: trypsin with at least 2 missed cleavages with 7 to 52 peptide length, charge states of +2, +3, and +4 accepted. Cysteine carbamidomethyl was set as a fixed modification, while oxidation (M), hydroxyproline, and acetyl on protein N-termini were set for variable modifications. All results were filtered at 1% false discovery rate, and protein quantitation was performed at the MS1 level. The data was normalized by retention time-dependent local regression model (1) and protein groups (PGs) were inferred by IDPicker. In this study, PGs are defined as one or more UniProt protein accessions that share identified peptide evidence and cannot be unambiguously distinguished by unique peptides and therefore are grouped and quantified together.

1.1.2.2 Quantification of protein relative abundances

Protein quantification pivot reports were exported from Spectronaut, and PGs were quantified using all peptides with the intensity-based absolute quantification (iBAQ) approach. The average iBAQ

value for each protein group was calculated as the sum of all identified values divided by the number of theoretical fully specific peptides without missed cleavages, as calculated in Spectronaut. Relative abundances were calculated by dividing each PG's average iBAQ value by the summed iBAQ value. Unquantified entries and entries below $\log_1$ scale were removed as they were regarded as "noise", retaining the remaining entries for subsequent analysis.

1.1.2.3 Allergenic proteins from allergen databases

For peanut, soybean, chickpea, winter pea, white bean, sweet blue lupine and beluga lentil, a curated database containing allergen accessions was created using information from four allergen databases: WHO/IUIS Allergen Nomenclature, Allergome, Comprehensive Protein Allergen Resource (COMPARE), and AllergenOnline. A Basic Local Alignment Search Tool (BLAST) was then performed for each accession present in the four allergen databases on UniProt BLAST against the respective species. The additional accessions obtained from BLAST were added to the database when they met the criteria of similarity ≥ 90%, score ≥ 100, and e-value $\leq 10^{-7}$. This study defines an allergen as protein accessions that are annotated as an allergen in at least one of the four allergen databases or identified through a 90% BLAST to the known allergen accessions. One accession does not represent a full allergen, but rather a fragment of the allergen (2). Normalized mean iBAQ values were summed to calculate the relative abundance of allergens of individual allergenic proteins relative to the proportion of all proteins.

1.1.2.4 Protein family naming framework to identify additional proteins belonging to allergenic protein families

To identify additional proteins belonging to allergenic protein families for both known and less-studied legumes with regards to allergenicity, a novel approach based on a protein family-based naming framework was developed. All protein families associated with the nine legume species with accepted allergens available in WHO/IUIS were first identified (Table S6). For each species, the corresponding species UniProt databases containing all protein entries were retrieved from UniProt. Keywords for identifying additional proteins belonging to each allergenic protein family were derived based on names of known allergen accessions from the databases coupled with 90% BLAST, and protein family annotations in UniProt (Table S7). The number of peptides, proteins, and protein groups identified for each of the legumes also reported (Table S8). The relative abundances of these proteins belonging to the corresponding allergenic protein families were summed as above, relative to the proportion of all proteins (Table S9).

2 Supplementary Tables

Table S1. sIgE levels of selected subjects with sensitization to legumes. Allergen-specific IgE antibodies are expressed as kU_A/L.

Source	Type	PEI 1	PEI 2	PEI 3	PEI 4	PEI 6	PEI 7	PEI 8	PEI 9	PEI 11	PEI 12	PEI 13	PEI 14
**Lup a**	E	4.6	3.3	6.6	7.2	0.4	6.1	0.1	0.3	2.5	1.2	< 0.1	0.6
**Pis s**	E	1.9	0.8	3.6	7.1	< 0.1	3.8	0.2	< 0.1	1.6	0.2	< 0.1	0.5
**Len c**	E	8.3	0.5	6.0	9.0	0.3	1.8	0.2	0.1	0.9	0.4	0.2	0.4
**Cic a**	E	6.9	1.6	0.9	6.2	1.8	3.9	0.2	0.3	1.1	0.6	0.2	0.7
**Tri fo**	E	1.5	1.0	0.8	4.7	0.2	1.3	0.1	0.2	1.3	4.2	0.2	0.4
**Ara h 1**	P	4.2	32.0	36.4	45.9	15.0	> 50	0.1	0.1	39.6	15.3	0.1	32.9
**Ara h 2**	P	< 0.1	43.9	4.3	46.7	10.3	> 50	< 0.1	1.4	44.6	45.9	< 0.1	39.2
**Ara h 3**	P	19.1	32.5	10.6	46.1	18.3	> 50	0.1	0.2	43.1	8.5	0.1	22.8
**Ara h 5**	P	< 0.1	0.2	1.5	1.9	< 0.1	3.1	< 0.1	< 0.1	2.4	6.6	< 0.1	0.5
**Ara h 6**	P	0.1	40.8	25.4	47.9	2.2	49.7	0.1	0.2	43.9	27.8	0.2	25.2
**Ara h 8**	P	< 0.1	< 0.1	9.0	2.1	< 0.1	1.3	0.2	0.8	31.6	3.6	8.0	19.1
**Ara h 9**	P	5.8	1.7	< 0.1	< 0.1	< 0.1	0.3	0.3	0.3	0.6	< 0.1	< 0.1	< 0.1

**Gly m 4**	P	< 0.1	< 0.1	0.2	1.2	< 0.1	0.5	< 0.1	< 0.1	< 0.1	0.9	< 0.1	0.7
**Gly m 5**	P	36.6	5.2	10.3	19.3	1.1	7.7	0.2	0.2	1.8	0.9	0.1	2.0
**Gly m 6**	P	40.7	5.9	1.8	35.1	2.6	7.9	< 0.1	0.1	7.1	3.9	0.1	2.1
**Gly m 8**	P	< 0.1	0.3	< 0.1	< 0.1	1.2	< 0.1	0.3	1.0	< 0.1	< 0.1	< 0.1	< 0.1
**Len c 1**	P	1.3	0.3	4.2	25.0	< 0.1	0.1	< 0.1	< 0.1	1.7	0.4	< 0.1	0.3
**Len c 3**	P	2.5	0.7	0.5	1.5	0.1	1.3	< 0.1	0.2	0.5	0.2	< 0.1	0.5
**Pis s 1**	P	1.0	0.7	0.8	25.1	< 0.1	3.5	< 0.1	< 0.1	1.1	0.4	< 0.1	0.7
**Pis s 2**	P	0.1	1.2	2.6	6.2	0.1	4.4	< 0.1	0.1	0.5	0.7	< 0.1	0.7
**Pis s 3**	P	0.4	0.3	0.1	< 0.1	0.2	< 0.1	< 0.1	< 0.1	2.3	< 0.1	< 0.1	< 0.1

Measuring range for sIgE is 0.3 – 50 kU_A/L (quantitative). E = extract, P = purified protein, < 0.1 = below limit of detection. Allergen abbreviations for allergen components (e.g. Ara h 1, Gly m 5) follow nomenclature of registered allergens according to IUIS/WHO (www.allergen.org), abbreviations for total protein extracts from legume seeds refer to lupine (Lup a), pea (Pis s), lentil (Len c), chickpea (Cic a), and fenugreek (Tri fo).

Table S2. Comparison of protein concentration of in-house legume seed extracts obtained using different buffers and calculated means for each extraction buffer.

Seed material	Protein concentration (mg/mL)			
	TBS pH 7.4	PBS pH 7.1	AC pH 9.0	SC pH 9.6
Roasted peanut	0.5	0.5	2.6	3.4
White lupine	6.7	5.5	7.1	7.2
Yellow lupine	3.4	2.3	9.4	9.4
Blue lupine	4.4	2.6	7.0	6.7
Yellow soybean	3.0	2.6	4.5	3.7
Pea	5.0	3.4	6.7	5.6
Green pea	5.9	3.3	6.5	5.3
Brown lentils	3.5	2.6	6.0	4.2
Red lentils	5.4	5.9	9.2	8.3
Chickpea	3.0	2.5	6.9	5.8
White bean	1.3	1.6	2.4	2.5
Kidney bean	1.4	2.3	3.3	2.3
Pinto bean	1.2	1.6	3.1	2.8

Table S3. Comparison of protein concentration from ALLPreT legume seeds extracted at ratios of 1:20 and 1:5 (w/v) using AC buffer under same extraction conditions.

ALLPreT Seed material	Protein concentration (mg/mL)	
	**1:20**	**1:5**
Roasted peanut	6.2	20.5
Soybean	7.5	35
Winter pea	6.0	20.6
Beluga lentils	6.1	25.4

Table S4. Comparison of protein concentration of ALLPreT legume seed extracts obtained using non-defatted and defatted seed powder for protein extraction with AC buffer.

Seed material	Protein concentration (mg/mL)	
	**Non-defatted**	**Defatted**
Roasted peanut	14.4	45.9
Unroasted peanut	23.3	22.3
Sweet blue lupine	33.8	34.6
Soybean	31.3	34.5
Winter pea	23.7	17.5
Beluga lentils	27.7	27.3
Chickpea	30.2	37.7

Field bean	42.6	53.6

Table S5. Protein concentration, extraction yield, and estimated extraction efficiency of legume proteins as calculated against selected reference protein content. Total protein extracted (%) refers to the amount of extractable protein relative to the initial seed weight.

Seed material	Protein concentration (mg/mL)	Yield: total protein extracted (%)	Reference protein content (%)	Estimated extraction efficiency (%)
Roasted peanut	16.4	8.2	24.4[a]	33.6
Unroasted peanut	37.8	18.9	23.3[a]	81.1
Sweet blue lupine	47.6	23.8	33.3[b]	71.5
Soybean	40.6	20.3	36.5[a]	55.6
Winter pea	22.7	11.4	23.1[a]	49.1
Beluga lentils	25.2	12.6	25.7[a]	49
Chickpea	27.3	13.7	20.5[a]	66.6
Field bean	32.5	16.3	26.1[a]	62.3
White bean	16.4	8.2	23.4[a]	35
Fenugreek	8.9	17.8	23[a]	77.4
Alfalfa	8.8	17.6	18.2[a]	96.7
Red clover	10.1	20.2	34.5[c]	58.6
White clover	8.5	17	27.7[d]	63

Range of estimated extraction efficiency (%)	34 - 97
Median of estimated extraction efficiency	62.3

The values of reference protein content are expressed as percentage (g/100 g*100). Reference values were obtained from food and feed composition databases or literature sources. [a]USDA FoodData Central (3), [b]INRAE–CIRAD–AFZ Feed Tables (4) and [c]Butkutė et al. (5) report crude protein calculated as nitrogen N × 6.25 based on Kjeldahl or Dumas total nitrogen determination. Values from [d]Elamine et al. (6) are based on total amino acid analysis and expressed as protein equivalents. Extraction efficiency (%) was calculated as the ratio between total protein extracted (%) and reference protein content (%), multiplied by 100.

Table S6. ALLPreT legume seeds: Identified allergens belonging to legume allergen-related protein families from the nine legumes with identified allergens in the WHO/IUIS allergen database (https://allergen.org/).

**Legume Protein family**	**Peanut (*Arachis hypogaea*)**	**Narrow-leaved blue lupine (*Lupinus angustifolius*)**	**Soybean (*Glycine max*)**	**Pea (*Pisum sativum*)**	**Lentil (*Lens culinaris*)**	**Chickpea (*Cicer arietinum*)**	**White lupine (*Lupinus albus*)**	**Green bean / White bean (*Phaseolus vulgaris*)**	**Mung bean (*Vigna radiata*)**
**11S globulin**	1	-	1	-	-	-	-	-	-
**7S globulin**	1	1	1	2	1	-	-	-	1
**2S albumin**	3	-	1	-	-	-	-	-	-
**Seed albumin**	-	-	-	-	-	-	-	-	1
**Pathogenesis-related 10 protein (PR-10)**	1	-	1	-	-	-	-	-	1
**Profilin**	1	-	1	-	-	-	1	-	-
**Non-specific lipid transfer protein (nsLTP)**	3	1	-	1	1	-	-	1	-

**Oleosin**	4	-	-	-	-	-	-	-	-
**Defensin**	2	-	-	-	-	-	-	-	-
**Cyclophilin**	1	-	-	-	-	-	-	-	-
**Annexin**	1	-	-	-	-	-	-	-	-
**Seed biotinylated protein (SBP)**	1	-	1	-	-	-	-	-	-
**Late embryogenesis abundant protein (LEA)**	-	-	-	-	-	1	-	-	-
**Cytokinin-specific binding protein (CSBP)**	-	-	-	-	-	-	-	-	1
**Total protein families**	**11**	**2**	**6**	**2**	**2**	**1**	**1**	**1**	**4**

Table S7. Keywords of UniProt protein entries identifying potential proteins belonging to legume allergen-related protein families (https://www.uniprot.org/).

Protein family	Keywords of "Protein names"
**11S globulin**	
Peanut	'Cupin type-1 domain-containing protein', 'Seed storage globulin', 'Arachin 6', 'Arachin 7', 'Arachin Arah3 isoform', 'Ara h 3 allergen', 'Glycinin', 'Iso-Ara h3', 'Arachin Ahy', 'Storage protein', 'Allergen Arah3', 'Gly1', potential additional – 11S arachin
Soybean	'Glycinin', 'Cupin type-1 domain-containing protein'
Chickpea	'Legumin', 'Glutelin'
Lentil	'Cupin type-1 domain-containing protein', 'Legumin'
Pea	'Cupin type-1 domain-containing protein', 'Legumin'
Field bean	'Legumin', 'Cupin type-1 domain-containing protein'
White bean	'Legumin', 'Cupin type-1 domain-containing protein'
Red clover	'Legumin', 'Glutelin type-A'
Alfalfa	'Glycinin'
White clover	'Legumin', 'glutelin type-A', Cupin type-1 domain-containing protein'
Fenugreek	'Legumin', 'Glycinin', 'Glutelin', 'Cupin type-1 domain-containing protein'
**7S globulin**	
Peanut	'Allergen Ara h 1', 'Allergen Ara h I', 'Conarachin'
Sweet blue lupine	'Conglutin beta', 'Cupin type-1 domain-containing protein', 'Conglutin gamma'
Soybean	'Beta-conglycinin', 'Cupin type-1 domain-containing protein'
Pea	'Cupin type-1 domain-containing protein', 'Vicilin'
Lentil	'Allergen Len c 1.01', Vicilin', 'Convicilin', 'Cupin type-1 domain-containing protein'
Chickpea	'Vicilin-like', 'Provicilin-like'
Field bean	'Cupin type-1 domain-containing protein', 'Vicilin', 'Convicilin'
White bean	'Phaseolin', 'Alpha-phaseolin',
Red clover	'Vicilin-like protein', 'Convicilin'
Alfalfa	'Cupin, RmIC-type', 'Cupin'
White clover	'Vicilin-like protein', 'Convicilin', Cupin type-1 do

Fenugreek	'Cupin type-1 domain-containing protein', 'Vicilin', 'Convicilin'
**2S albumin**	
Peanut	Ara h 2: 'Conglutin-7', 'Ara h 2 allergen'<br>Ara h 6: 'Conglutin', 'Bifunctional inhibitor/plant lipid transfer protein/seed storage helical domain-containing protein', 'Conglutin 8', 'Ara h 6'<br>Ara h 7: 'Bifunctional inhibitor/plant lipid transfer protein/seed storage helical domain-containing protein', 'Conglutin', 'Ara h 7 allergen', '2S protein 2'
Sweet blue lupine	'Conglutin delta 1', 'Bifunctional inhibitor/plant lipid transfer protein/seed storage helical domain-containing protein'
Soybean	'2S seed storage albumin protein', 'Napin-type 2S albumin 1', 'Bifunctional inhibitor/plant lipid transfer protein/seed storage helical domain-containing protein',
Chickpea	'2S albumin-like'
Fenugreek	'2S albumin seed storage protein'
Alfalfa	'2S albumin seed storage protein'
Red clover	'Conglutin'
White clover	'Conglutin'
**Seed albumin**	
Pea	'Albumin-1', 'Albumin-2', 'Seed albumin'
Lentil	'Albumin I', 'Albumin-1', 'Albumin-2'
Chickpea	'Albumin-2-like'
Field bean	'Albumin I'
White bean	'Albumin-1', 'Albumin-2'
Fenugreek	'Albumin-2'
Alfalfa	'Albumin-2 protein'
Red clover	'Albumin', 'Albumin-2'
White clover	'Albumin I'
**Pathogenesis-related 10 protein (PR-10)**	
Peanut	'Bet v I/Major latex protein domain-containing protein', 'Pathogenesis-related class 10 protein', 'Pathogenesis-related protein 10', 'Ara h 8 allergen'

Soybean	'Bet v I/Major latex protein domain-containing protein', 'Stress-induced protein SAM22', 'G.max mRNA from stress-induced gene (H4)'
Red clover	'Pathogenesis-related protein pr10', 'Major allergen Pru ar 1-like protein', 'Aba-responsive protein abr17'
Other legumes	'Pathogenesis-related protein 10'
**Profilin**	
All legumes	'Profilin'
**Non-specific lipid transfer protein (nsLTP)**	
Peanut	'Non-specific lipid-transfer protein', 'Bifunctional inhibitor/plant lipid transfer protein/seed storage helical domain-containing protein'
Other legumes	'Non-specific lipid-transfer protein'
**Oleosin**	
All legumes	'Oleosin'
**Defensin**	
Peanut	'Defensin 1 (allergen Ara h 12)', 'Defensin 2 (allergen Ara h 13.0101)', 'Defensin 3 (allergen Ara h 13.0102)' 'Knottin scorpion toxin-like domain-containing protein'
Other legumes	'Knottin scorpion toxin-like domain-containing protein'
**Cyclophilin**	
All legumes	'Peptidyl-prolyl cis-trans isomerase'
**Annexin**	
All legumes	'Annexin'
**Seed biotinylated protein (SBP)**	
Peanut and other legumes	'Seed biotin-containing protein SBP65'
Soybean	'Seed biotin-containing protein SBP65', 'Seed biotinylated protein', 'LEA protein'
**Late embryogenesis abundant protein (LEA)**	
All legumes	'Late embryogenesis abundant protein'
**Cytokinin-specific binding protein (CSBP)**	
Mung bean	'Phytohormone-binding protein CSBP (Cytokinin-specific binding protein) (VrCSBP) (Major pollen allergen Bet v 1-like protein) (Pathogenesis-related PR10-like protein) (allergen Vig r 6)'
**Lectin**	

Peanut and other legumes	'Lectin'
White bean	'Lectin', 'Phytohemagglutinin'

Table S8 Total number of UniProt accession entries, peptides, proteins, and protein groups (PGs) identified in ALLPreT legumes using Spectronaut after iBAQ using all peptides against taxonomy database searches. The FASTA database files for all legumes were assessed in UniProt on 8 January 2026.

Legume	Accession entries in UniProt	No. of peptides identified	No. of proteins identified	No. of protein groups
Roasted peanut	102020	2636	1423	667
Unroasted peanut	102020	15324	6008	3145
Sweet blue lupine	31389	22090	4806	4301
Soybean	85130	18945	6408	3546
Pea	66517	22859	5168	3496
Lentil (searched against Fabeae family, Taxon ID 163743)	107132	16217	7227	3825
Chickpea	31239	29518	5192	4311
Field bean	36660	18689	3664	3097
White bean	32796	34735	6213	5421
Fenugreek (searched against **Trifolieae** Tribe, Taxon ID 163742)	311473	17041	10018	4731

Alfalfa (searched against ***Medicago*** genus, Taxon ID 3877, 94640 entries)	94640	27045	8845	5069
Red clover	60478	22012	5793	5105
White clover (searched against *Trifolium* genus, Taxon ID 3898)	215542	19780	8022	5351

Table S9. Relative abundance of all proteins belonging to legume allergen-related protein families or lectins in ALLPreT legumes based on a protein naming framework approach.

Protein family	Roasted peanut	Unroasted peanut	Sweet blue lupine	Soybean	Winter pea	Beluga lentil	Chickpea	Field bean	White bean	Fenugreek	Alfalfa	Red clover	White clover
**11S globulin**	58.9	21.3	21.5	29.5	16.7	22.2	22.2	44.8	4.8	7.8	18.6	21.7	30.3
**7S globulin**	17.2	11.7	30.7	9.1	27.9	37.9	8.1	16.2	3.6	24.2	8.5	41.6	20.8
**2S albumin**	13.4	40.1	8.1	5.3	-	-	0.3	-	-	1.7	21.1	1.0	< 0.1
**Seed albumin**	-	-	-	-	16.4	1.4	5.7	3.9	0.6	< 0.1	< 0.1	< 0.1	< 0.1
**PR-10**	0.1	0.6	0.1	0.1	0.4	< 0.1	< 0.1	0.1	0.1	0.1	< 0.1	< 0.1	< 0.1
**Profilin**	< 0.1	< 0.1	< 0.1	< 0.1	0.1	< 0.1	0.1	< 0.1	0.1	0.1	0.1	< 0.1	0.1
**nsLTP**	0.1	1.2	1.4	0.1	0.5	0.5	0.3	0.3	0.4	0.1	0.5	0.3	0.2
**Oleosin**	0.3	1.7	0.6	1.2	0.1	< 0.1	1.4	< 0.1	0.3	1.0	0.7	0.8	1.0
**Defensin**	0.2	1.4	0.5	0.2	1.5	0.4	0.5	1.9	3.3	1.8	0.9	-	< 0.1
**Cyclophilin**	< 0.1	0.3	0.2	0.2	0.3	0.3	1.4	0.2	0.4	0.8	0.4	0.4	0.7
**Annexin**	< 0.1	0.5	0.5	0.2	0.5	0.1	0.1	0.2	0.4	0.2	0.2	0.1	0.2
**SBP**	0.1	0.1	0.4	0.2	0.9	0.4	0.9	0.7	0.2	0.2	0.1	0.4	0.8
**LEA**	4.6	1.8	0.5	0.9	1.2	0.3	2.3	0.5	0.6	3.6	1.8	1.4	0.9
**CSBP**	-	-	-	-	-	-	-	-	-	-	-	-	-
**Lectin**	0.1	1.2	< 0.1	0.8	0.1	6.9	1.0	0.4	11.6	0.1	0.1	< 0.1	< 0.1

**% of proteins already identified as allergens from curated database**	87.9	76.3	46.8	42.4	23.9	21.7	10.0	-	0.3	-	-	-	-
**% of proteins belonging to allergen-related protein families**	94.9	80.7	64.6	47.0	66.5	63.6	43.3	68.8	14.8	41.6	52.9	67.8	55.1
**% of proteins not belonging to allergen-related protein families**	5.1	19.3	35.4	53.0	33.5	36.4	56.7	31.2	85.2	58.4	47.1	32.2	44.9

Table S10 Endotoxin level of ALLPreT study legume extracts after lyophilization and restorage in pure water. Results refer to endotoxin (EU/mL) at 1 mg/mL of protein.

**Seed name**	**Scientific name**	**EU/mL at 1 mg/mL**
Peanut roasted	*Arachis hypogaea*	3.2
Peanut unroasted	*Arachis hypogaea*	< 1.0
Sweet lupine blue	*Lupinus angustifolius*	2.6
Soybean	*Glycine max*	1.2
Beluga lentil	*Lens culinaris*	4.2
Winter pea	*Pisum sativum*	16
Chickpea	*Cicer arientinum*	< 1.00
Field bean	*Vicia faba*	38
White bean	*Phaseoulus vulgaris*	6.7
Fenugreek	*Trigonella-foenum graecum*	67
Alfalfa	*Medicago sativa*	33
Red clover	*Trifolium pratense*	318
White clover	*Trifolium repens*	351

3 Supplementary Figures

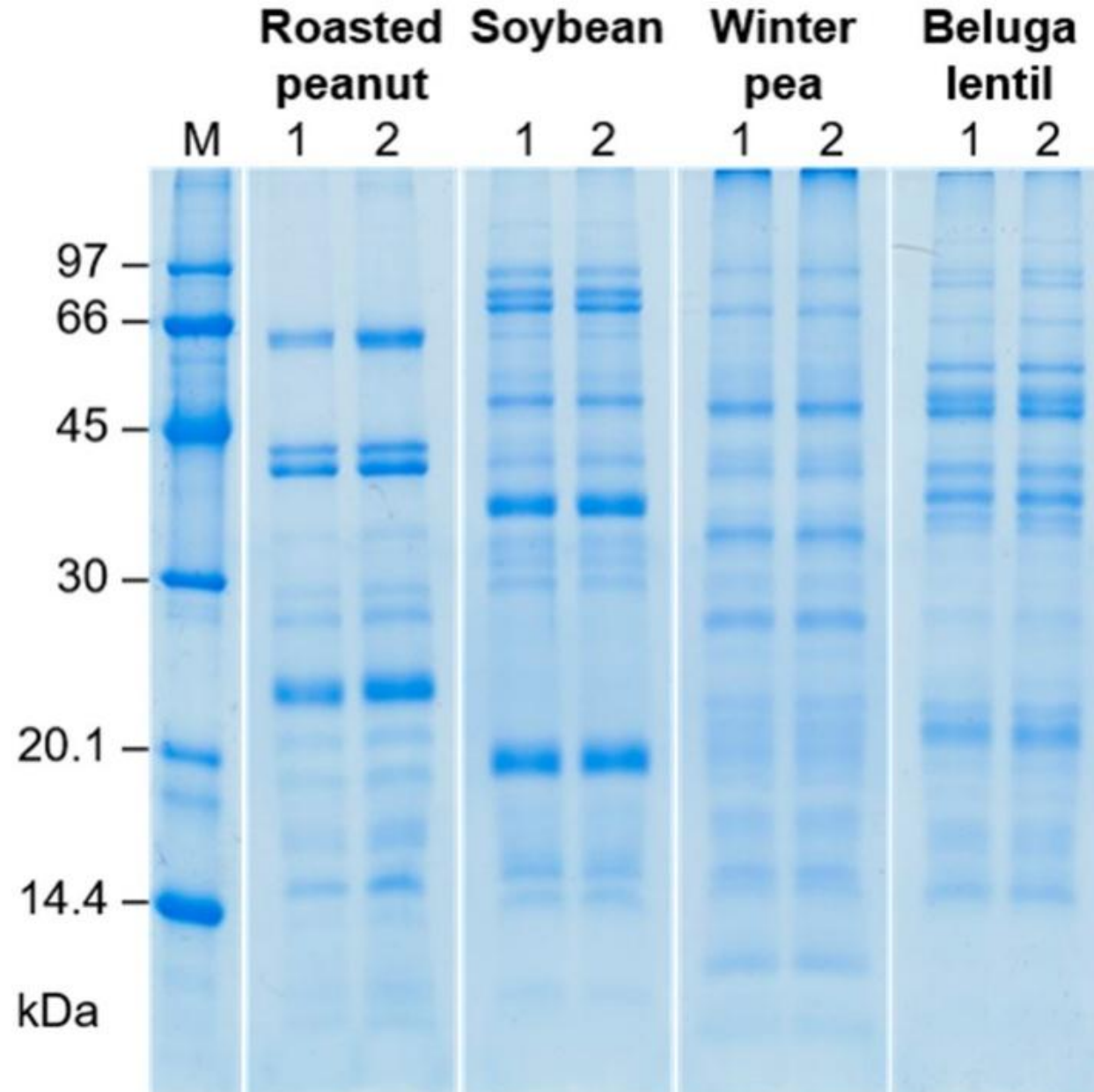

Figure S1. Protein profile of ALLPreT legumes seed protein extracted at 1:20 (lane 1) and 1:5 (lane 2) ratio (w/v) with AC buffer and analyzed at 10 µg/cm by SDS-PAGE under reducing conditions.

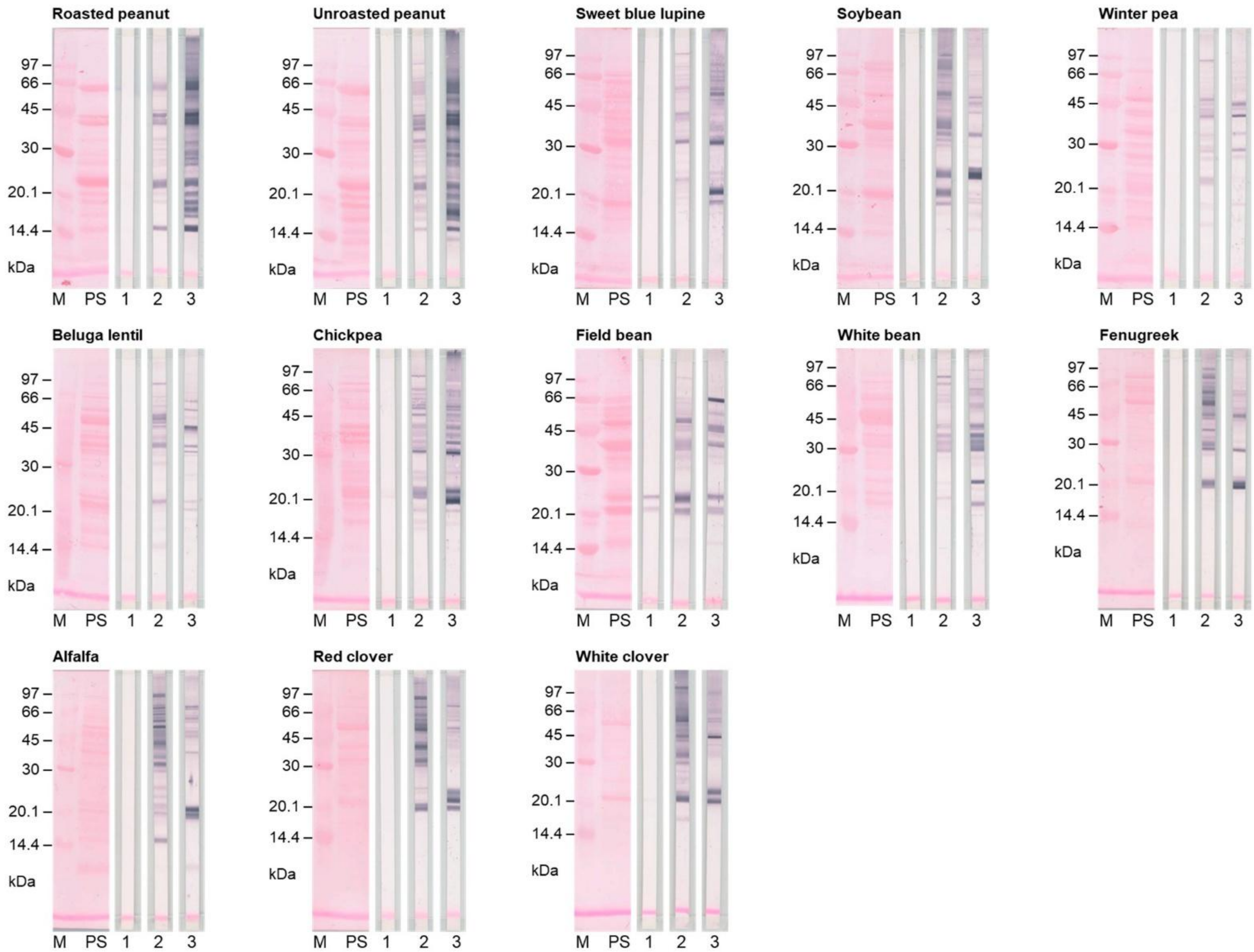

Figure S2. IgG immunoblot analysis of ALLPreT legumes seed protein extracts. Protein extracts were detected with IgG pAb to assess antibody recognition and cross-binding among legume species. M = marker, PS = Ponceau S staining of whole extract, 1 = buffer control (anti-rabbit IgG), 2= anti-soybean (dilution 1:100,000 or 1:25,000), 3 = anti-peanut (dilution 1:10,000).

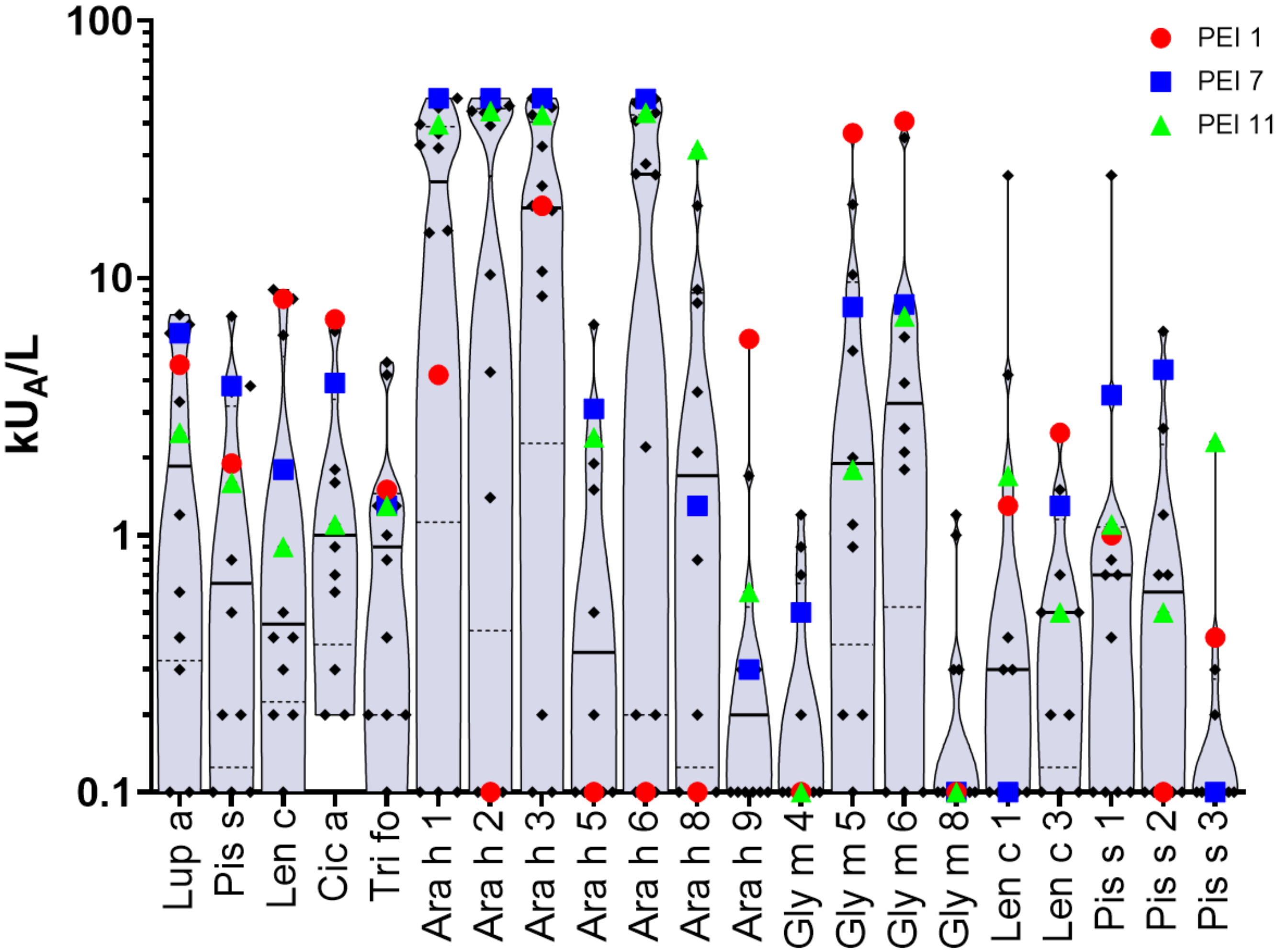

Figure S3. Allergen-specific IgE levels (kU_A/L) in the analyzed human sera samples shown as a violin plot. The violin shape represents the distribution density of values, the central line indicates the median, and the dashed lines indicate the interquartile range. Each dot represents one individual serum sample (n = 12).

4 References

1. Callister SJ, Barry RC, Adkins JN, Johnson ET, Qian WJ, Webb-Robertson BJM, Smith RD, Lipton MS. Normalization approaches for removing systematic biases associated with mass spectrometry and label-free proteomics. *J Proteome Res* (2006) 5:277–286. doi: 10.1021/pr050300l
2. Krutz NL, Kimber I, Winget J, Nguyen MN, Limviphuvadh V, Maurer-Stroh S, Mahony C, Gerberick GF. Identification and semi-quantification of protein allergens in complex mixtures using proteomic and AllerCatPro 2.0 bioinformatic analyses: a proof-of-concept investigation. *J Immunotoxicol* (2024) 21:2305452. doi: 10.1080/1547691X.2024.2305452
3. U.S. Department of Agriculture ARS. FoodData Central. *Beltsville Human Nutrition Research Center* https://fdc.nal.usda.gov/. [Accessed January 7, 2026]

4. INRAE CA. INRAE-CIRAD-AFZ Feed tables. https://www.feedtables.com/ [Accessed January 7, 2026]
5. Butkute B, Taujenis L, Norkevičien˙ EE, Wang M, Zhao Y. Small-Seeded Legumes as a Novel Food Source. Variation of Nutritional, Mineral and Phytochemical Profiles in the Chain: Raw Seeds-Sprouted Seeds-Microgreens. *Molecules* (2018) doi: 10.3390/molecules24010133
6. Elamine Y, Alaiz M, Girón-Calle J, Guiné RPF, Vioque J. Nutritional Characteristics of the Seed Protein in 23 Mediterranean Legumes. *Agronomy* (2022) 12:400. doi: 10.3390/agronomy12020400
